# Fyn inhibition by TAE684: A synergistic strategy to suppress melanoma and reverse vemurafenib resistance

**DOI:** 10.1038/s41419-025-08090-1

**Published:** 2025-11-06

**Authors:** Waner Liu, Xu Zhang, Xiaowei Liang, Yeye Guo, Zhe Zhou, Susi Zhu, Cong Peng, Xiang Chen

**Affiliations:** 1 The Department of Dermatology, Xiangya Hospital, Central South University, Changsha, Hunan China; 2 Furong Laboratory, Changsha, Hunan China; 3 Hunan Key Laboratory of Skin Cancer and Psoriasis, Xiangya Hospital, Central South University, Changsha, Hunan China; 4 Hunan Engineering Research Center of Skin Health and Disease, Xiangya Hospital, Central South University, Changsha, Hunan China; 5 National Clinical Research Center for Geriatric Disorders, Xiangya Hospital, Central South University, Changsha, Hunan China

**Keywords:** Melanoma, Drug development, Melanoma, Cancer therapeutic resistance, Targeted therapies

## Abstract

Therapies targeting BRAF can inhibit the development of melanoma with BRAF mutations and enhance survival rates, though acquired resistance inevitably arises. The non-receptor tyrosine kinase Fyn, recognized for its role in regulating tumor cell survival and drug resistance, has emerged as a promising therapeutic target in melanoma treatment. In this study, we conducted a virtual screening and identified TAE684 as a potent inhibitor of Fyn. Utilizing in vitro assays, including assessments of cell viability, reactive oxygen species (ROS) production and DNA damage, alongside an in vivo melanoma xenograft model, we demonstrated that either TAE684 treatment or Fyn knockdown resulted in increased ROS levels and DNA damage, ultimately inducing cell cycle arrest at the G2/M phase and apoptosis in melanoma cells. Significantly, the application of TAE684 in melanoma cells demonstrated a capacity to counteract vemurafenib resistance, presumably through the down-regulation of the AP-1 pathway. Furthermore, the combination of TAE684 with vemurafenib exhibits a synergistic effect, leading to decreased cell viability in melanoma cells resistant to vemurafenib treatment. These results highlight the potential of TAE684 as a dual-function agent that not only inhibits melanoma proliferation but also reverses resistance to vemurafenib by targeting Fyn, thereby establishing it as a promising candidate for melanoma therapy.

## Introduction

Melanoma is presently the most aggressive type of skin cancer, with steadily rising incidence and mortality rates for the past 20 years [1–3]. Although early-stage melanoma can be successfully managed with surgical treatment, its survival rate significantly decreases after metastasis [4], prompting extensive efforts to identify effective therapeutic strategies for melanoma. Currently, clinical approaches for metastatic melanoma primarily include immunotherapy and targeted therapy [5]. Immunotherapy has demonstrated superior efficacy in advanced melanoma and significantly improved overall survival [5, 6]. Nonetheless, many patients have a low response immunotherapy [7]. The high cost and severe side effects also limit its application [8]. By contrast, targeted therapy generally exhibits high initial response rates and has been a mainstream anti-cancer therapeutic modality, although resistance frequently develops within months and leading to recurrence [9, 10]. Thus, finding ways to conquer acquired resistance to targeted therapies is critical for improving the outcome of melanoma patients.

One of the earliest and most frequent genetic changes recognized in human melanoma is the BRAF mutation, occurring at a rate of 40.3% [11, 12]. Mutated BRAF constitutively activates the MAPK pathway, promoting the expression of various downstream oncogenes and facilitating tumorigenesis and progression [13]. BRAF V600E, the most frequently observed BRAF mutation, enhances BRAF activity by ~700 times [11]. Targeted BRAF inhibitors (BRAFi) such as vemurafenib and dabrafenib are vital therapeutic options for melanoma patients carrying BRAF V600E mutation and demonstrate significant therapeutic efficacy [14, 15]. However, resistance to BRAFi often develops shortly after initial treatment primarily due to aberrant reactivation of the MAPK pathway [16].

Combining BRAFi with MAPK inhibitors has shown improved and more durable clinical outcomes [17]. Nonetheless, nearly all patients receiving these combination therapies experience adverse events [18], and acquired resistance typically develops within a median of 9–11 months [19], presenting an ongoing challenge. A number of small molecule drugs have been created and demonstrated promise in treating BRAFi resistant cells. Histone deacetylase inhibitors, when used with BRAFi, can increase its antitumor effect and delay the development of BRAFi acquired resistance [20, 21]. The combination of compound C012 and vemurafenib enhances TRIM16 protein expression and decreases melanoma cell viability in both laboratory and live models [22]. Hence, further investigation of potential drug candidates for combination with BRAFi offers a viable approach to addressing BRAFi resistance in melanoma.

Fyn, a non-receptor tyrosine kinase, is associated with the regulation of cell growth, apoptosis, morphological transformation, and motility [23]. It has been found to be highly expressed in cancers such as melanoma, where it is associated with poor prognosis and reduced survival [24]. Studies have explored the contribution of Fyn to drug resistance. In breast cancer, Fyn confers tamoxifen resistance through activating key cell cycle-related proteins [25]. Excessive activation of Fyn promotes resistance development in dasatinib-resistant chronic myeloid leukemia cells [26]. Fyn also regulates imatinib resistance in prostate cancer via interactions with miR-128/193a-5p/494 [27], and phosphorylated Fyn plays a crucial role in the resistance to vinorelbine in non-small cell lung cancer [28]. In this study, we found that vemurafenib resistance in melanoma cells can be reversed by inhibiting Fyn through either TAE684 treatment or gene knockdown. TAE684 induces apoptosis in melanoma cells by causing DNA damage. Meanwhile, the combination of TAE684 and vemurafenib exhibits a synergistic effect, offering insights into sensitizing melanoma to BRAF-targeted therapy.

## Methods and materials

### Cell lines and cell culture

Human melanoma cell lines SK-MEL-5, SK-MEL-28, and A375 along with human kidney cell line HEK293T were purchased from ATCC and employed adhering to the methodologies outlined in a prior investigation [29]. All cell lines were cultured in DMEM (11965092; Gibco, CA, USA) with 10% fetal bovine serum (FBS; ExCell Bio, Shanghai, China) and 1% penicillin-streptomycin (03-031-1B; Biological Industries, Beth Haemek, Israel), and incubated at 37 °C in a 5% CO_2_ environment. The vemurafenib-resistant A375 cells were generated as outlined before and maintained with 2.5 μM vemurafenib [30]. All cell lines have been authenticated by STR profiling and tested negative for mycoplasma contamination using mycoplasma detection kit (40612ES25, Yeasen, Shanghai, China).

### Antibodies

This study employed the following antibodies: anti-phosphotyrosine (P3300, Merck, Darmstadt, Germany); anti-Ki67 (ab15580; Abcam, MA, USA); anti-Lamin A/C (sc-376248; Santa Cruz, CA, USA). Meanwhile, anti-CD147 (11989-1-AP), anti-c-Fos (66590), anti-Bcl2 (12789) and anti-GAPDH (60004) were purchased from Proteintech (IL, USA). Anti-Fyn (4023), anti-p-Histone H2A.X (9718), anti-p53(2527), anti-phospho-p53 (Ser33) (2526), anti-p21 (2947), anti-c-Jun (9165), anti-cleaved PARP (9542) and anti-Bax (5023) were from Cell Signaling Technology (CST; MA, USA).

### In vitro pull-down assay

The binding of TAE684 (HY-10192, MedChemExpress, Shanghai, China) to Sepharose 4B beads was performed according to previously outlined methods [31]. About 1.5 mg of protein was incubated with TAE684-Sepharose 4B, gently shaken overnight at 4 °C in an incubation buffer. Subsequently, rinsed the beads five times with a washing buffer. The protein of interest was analyzed through immunoblotting.

### In vitro kinase assay

To investigate the capacity of TAE684-treated Fyn to phosphorylate CD147, recombinant CD147 protein (82909-7-RR, Proteintech) was subjected to Fyn kinase (ab84696, Abcam) in a reaction buffer (CST) containing 0.1 mM ATP. After a 30-min incubation at 30 °C, phosphorylated CD147 was evaluated using specific antibodies.

### Protein extraction and immunoblot procedures

After lysing cells by RIPA buffer, the concentrations of proteins were quantified using a BCA Protein Assay Kit (CW0014S, CWBiotech, Beijing, China). For the isolation of nuclear proteins, NE-PER Nuclear and Cytoplasmic Extraction Reagents (78835; Thermo Scientific, MA, USA) were employed, following the recommended protocols from the manufacturer. Following SDS-PAGE separation, the resultant proteins were moved onto polyvinylidene fluoride membranes (Millipore, MA, USA) and incubated with specific antibodies. Protein bands were visualized using the Image Lab software, and band intensities were quantified by grayscale analysis using ImageJ. Target protein expression levels were normalized to control groups for densitometric analysis.

### Cell proliferation assay

Cells were plated into 96-well plates at a density of 3,000 cells per well and incubated for the specified duration (*n* = 6). Following the incubation period, cell counting kit-8 (CCK-8) solution (Selleck, TX, USA) was introduced, and the mixture was incubated according to the manual. The absorbance of the samples was then measured at 450 nm using a BioTek microplate reader and imager software (BioTek Instruments, VT, USA).

### Colony formation assay

Cells were seeded into 6-well plates at a density of 1,000 cells per well and subjected to treatment with different concentrations of TAE684 or vemurafenib (HY-12057, MedChemExpress) for 48 h, with DMSO serving as the control (*n* = 3). After treatment, the medium was discarded and replaced with DMEM every other day over a 2 week period. Following this, colonies were fixed using fixation solution, stained with crystal violet (Beyotime, Shanghai, China), and subsequently analyzed for colony count using ImageJ software [32].

### Wound healing assay

Cells were cultured in a 6-well plate at a density of 1 × 10^6^ cells per well (*n* = 6). A linear wound was made in the cell monolayer using a pipette tip. Following this, the cells were washed three times by PBS and then refreshed with DMEM containing 2% FBS and specific concentrations of TAE684. Images of the same field of view were captured every 24 h until the wound in the control group had fully healed. The unhealed wound area in the center of each image was quantified using ImageJ software and compared to the wound area at 0 h. The wound healing rate was calculated using the following formula: Wound healing rate (%) = (1 - wound area at 72 h / wound area at 0 h) × 100%. Statistical analysis was performed based on these quantifications.

### Transwell assay

The 8-μm pores Transwell chambers (Corning, NY, USA) were placed in a 24-well plate (*n* = 3). Matrigel (BD Biosciences, NJ; at a dilution ratio of 1:7 in serum-free DMEM) was put into chambers and left to solidify. Cells were diluted in serum-free medium at a density of 5 × 10^4^ cells/0.1 mL and added into the upper chambers. Meanwhile, 0.5 mL DMEM with 30% FBS was introduced to the lower chamber. After 24 h or 48 h, cells were fixed using fixation solution and stained with crystal violet. Invaded cells on the bottom side were counted with an inverted microscope and quantified using ImageJ software, with three fields counted per well.

### Xenograft model

The animal research was approved by the Ethics Committee of Xiangya Hospital, Central South University (Changsha, China). 5 week-old female BALB/c nude mice were purchased from the Central South University Laboratory Animal Department. 5 × 10^6^ SK-Mel-28 or 2 × 10^6^ RA cells in 100 μL cold serum-free DMEM was transplanted through subcutaneous injection on the right flank of mice. After injection, mice were randomly assigned to different treatment groups. A sample size of 6 animals per group was chosen based on previous studies to ensure sufficient power and reliable results. The size of tumor was measured every 2 days by calipers, with its volume calculated as length × width × width × 0.5. Blinding was performed during assessment. The tumor tissues were removed, preserved in 10% formalin, and embedded in paraffin for subsequent immunohistochemical analysis.

### Immunohistochemistry

Paraffin-embedded tumor slices were prepared following the previously described method [29]. The antibody against Ki67, and c-Jun, c-Fos was utilized in this assay. Positive Ki67 and c-Jun staining photo was captured by randomly selecting and analyzed by ImageJ software (*n* = 3).

### Comet assay

The comet assay was completed in accordance with the instructions (C2041M, Beyotime). Briefly, cells were pre-treated with TAE684 for 48 h or infected with lentivirus. Then 1500 cells in each group were collected and blended with 65 μL agarose, then spread onto special comet slides and solidified. The cells underwent lysis at 4 °C, and electrophoresis was conducted using a chilled alkaline buffer for 20 min. The slides were washed in 0.4 M Tris-HCl buffer for 5 min, and were stained with propidium iodide. Fluorescent photographs were obtained using a fluorescence microscope. The CASP software was used to evaluate the tail moment (*n* = 8).

### Immunofluorescence

The immunofluorescence analysis was conducted following the previously outlined method [33]. Briefly, after cells were seeded on coverslips, 0.8 μM TAE684 in DMEM was added and cultured for 48 h (*n* = 3). For Fyn knocked down melanoma cells they are seeded on coverslips and cultured overnight. The coverslips carrying cells were rinsed with PBS and fixed with fixation solution. Then, cells were blocked by 5% BSA supplemented with 0.05% Triton-X 100 and covered with anti-γH2A.X antibody at 4°C for 16 h. Cells were washed and covered by the second antibody for 1 h at room temperature, and stained with DAPI (AiFang biological, Changsha, China) for 15 min. Fluorescent photographs were obtained using a fluorescence microscope.

### Apoptosis analysis and cell cycle analysis

In the apoptosis assay, cells pre-treated with designated concentrations of TAE684 for 48 h or cells with Fyn knockdown were stained with a pair of fluorescent dyes (FITC-Annexin V and PI) (*n* = 3). In the cell cycle assay, the pre-treated cells were fixed using chilled 70% ethanol and then stained with PI for 20 min at room temperature, keeping them away from light. Cells were subjected to flow cytometry (LSRFortessa, BD Biosciences). FlowJo software was used to perform data analysis.

### RNA-sequencing (RNA-seq) and analysis

SK-MEL-28 cells, either untreated or treated with 0.8 μM TAE684 for 48 h, were collected and sent to BGI (Shenzhen, China) for RNA-seq. Differential gene expression analysis was performed using the BGI online platform Dr. TOM (https://biosys.bgi.com/), with Gene Ontology (GO) biological process enrichment analysis conducted through the same platform. Gene Set Enrichment Analysis (GSEA) and visualization were performed at the Xiantao platform (https://www.xiantaozi.com/), based on ggplot2 (version 3.4.4).

### Statistical analysis methods

Statistical analyses were performed using GraphPad Prism 9.0. For comparisons between two groups, unpaired two-tailed Student’s *t*-test was used. For comparisons among multiple groups, one-way ANOVA followed by Tukey’s post-hoc test was applied. Homogeneity of variances was assessed using Levene’s test. A *p*-value < 0.05 was considered statistically significant. Experiments were performed in triplicate. Statistical results are displayed as means ± standard deviations (s.d.).

Detailed descriptions of other experimental procedures, including virtual screening and molecular docking, reactive oxygen species (ROS) measurement, quantitative reverse transcription-PCR analysis, ADP-Glo kinase assay, electrophoretic mobility shift assays (EMSA), lentiviral packaging and cell infection, and dual-luciferase reporter gene assays, are provided in the Supplementary Materials.

## Result

### TAE684 effectively targeted and inhibited Fyn’s kinase activity in melanoma cells

**Fig. 1:**
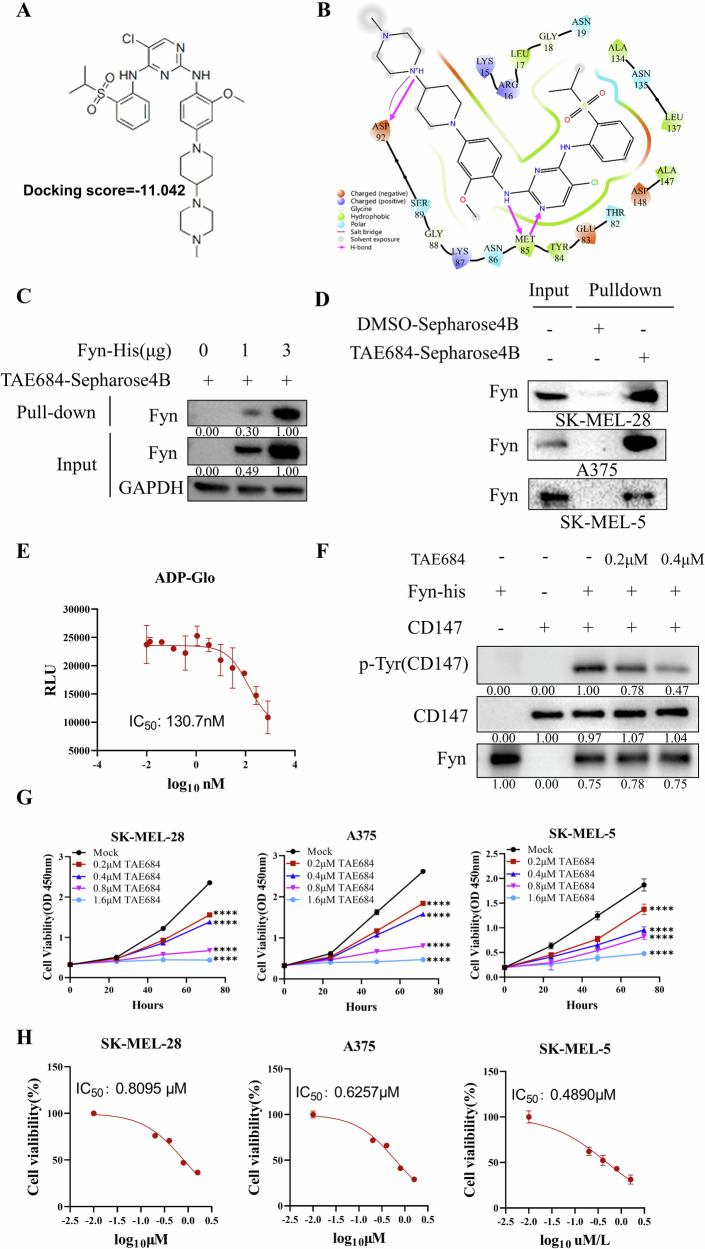
TAE684 prevents melanoma cells from proliferation via targeting Fyn. **A** The structure of TAE684. **B** The 2D protein-ligand interaction diagram of TAE684 with Fyn kinase showing binding interactions with amino acids. **C** HEK293T cells transfected with 0 μg, 1 μg, and 3 μg Fyn plasmid were lysed and incubated with TAE684-Sepharose4B. The combination of Fyn was detected by immunoblotting. **D** Lysates of melanoma cell lines, relatively SK-MEL-28, A375 and SK-MEL-5 cells, were incubated with TAE684-Sepharose4B or DMSO-Sepharose4B for pull-down assays. Anti-Fyn antibody was utilized to detect the combination. **E** A cell-free ADP-glo kinase assay was used to detect Fyn activity in vitro. The IC_50_ against Fyn of TAE684 was 130.7 nM. **F** In vitro kinase assays of Fyn activity with TAE684 in 0.2 μM and 0.4 μM. Reaction mixtures were subjected to immunoblot analysis using antibodies to p-Tyr, CD147, and Fyn. **G**, **H** CCK-8 OD values at 450 nm of SK-MEL-28, A375 and SK-MEL-5 cells treated with 0 μM, 0.2 μM, 0.4 μM, 0.8 μM, and 1.6 μM TAE684 at 0 h, 24 h, 48 h, and 72 h (*n* = 6). The value of IC_50_ was calculated by GraphPad Prism 9. The significance of differences was evaluated using one-way ANOVA. ****p < 0.0001.

Our previous study demonstrated that amodiaquine, an FDA-approved antimalarial drug, exhibits potent Fyn inhibitory activity with an IC_50_ of ~5 μM in SK-MEL-28 melanoma cells. To identify more potent Fyn inhibitors, a structure-based virtual screening was conducted using the chemical scaffold of amodiaquine (Fig. S1A) and the crystal structure of Fyn (Fig. S1B). This approach yielded 36 amodiaquine analogs with predicted Fyn-binding potential, hereafter referred to as FYN001–FYN036 (Fig. S1C). Among the screened compounds, TAE684 (FYN034) exhibited the most pronounced inhibitory effect on SK-MEL-28 cell proliferation at both 1 μM and 10 μM, as determined by cell viability assays (Fig. S2A, B). Furthermore, its favorable molecular docking score (-11.042) further supported its strong potential for Fyn binding, warranting its selection for further investigation (Fig. 1A). Docking analysis revealed that TAE684 binds to Fyn’s active site, forming hydrophobic interactions with ALA134, ASN135, LEU137, ALA147, and THR82, as well as hydrogen bonds with MET85 and ASP92(Fig. 1B). This drug-protein interaction was further confirmed through pull-down assays in both HEK293T and human melanoma cells (Fig. 1C, D). The inhibition of TAE684 on Fyn kinase activity was also validated in a cell-free ADP-glo kinase assay, revealing an IC_50_ of 130.7 nM (Fig. 1E). Since we previously reported that CD147 serves as a substrate for Fyn, the phosphorylation of CD147 was detected in an in vitro kinase assay after treating Fyn kinase with TAE684, and a concentration-dependent decrease was observed in the phosphorylation level (Fig. 1F). We then treated human melanoma cell lines SK-MEL-28, A375 and SK-MEL-5 with different concentrations of TAE684. TAE684 inhibited the proliferation of these cell lines in a dose-dependent manner, with IC_50_ values of 0.8095 μM in SK-MEL-28, 0.6257 μM in A375, and 0.4890 μM in SK-MEL-5 (Fig. 1G, H). TAE684 was originally identified as an ALK inhibitor [34]. However, treating melanoma cells with the ALK-selective inhibitor lorlatinib had no significant effect on proliferation, suggesting that TAE684 acts independently of ALK in melanoma (Fig. S3A).

### TAE684 suppressed the malignant phenotype of melanoma cells

**Fig. 2:**
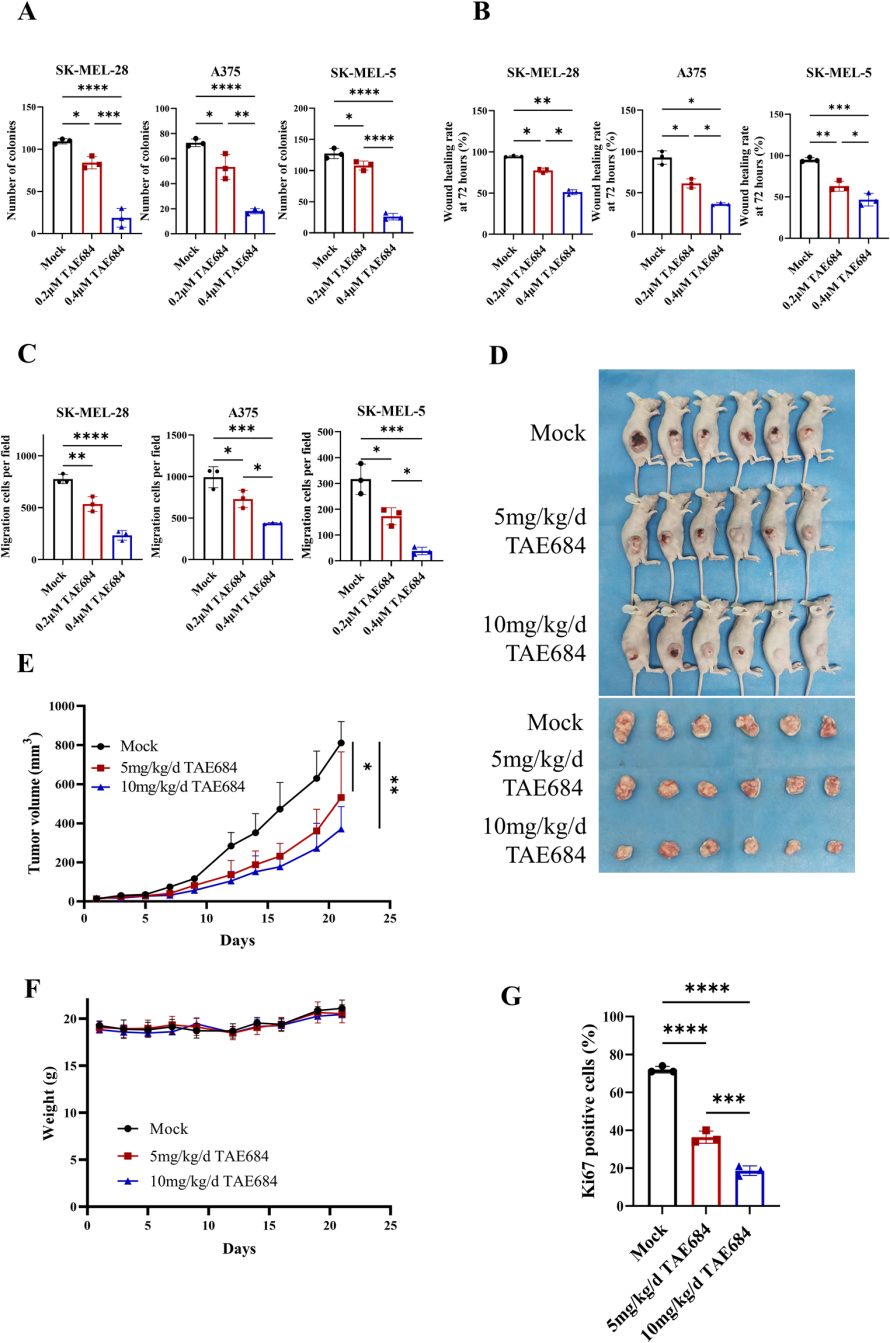
TAE684 suppresses the malignant phenotype of melanoma cells. **A** Colony formation assay to detect the number of SK-MEL-28, A375 and SK-MEL-5 cell clones at different concentrations (0 μM, 0.2 μM, 0.4 μM) of TAE684. The clones were stained with crystal violet and counted with image J software (*n* = 3). **B** Wound healing assays were performed to assess the invasion ability of SK-MEL-28, A375 and SK-MEL-5 cells treated with different concentrations (0 μM, 0.2 μM, 0.4 μM) of TAE684. The cell wound area at 72 h was analyzed using ImageJ software, and the wound healing rate was statistically analyzed (*n* = 3). **C** In transwell assays, SK-MEL-28, A375 and SK-MEL-5 cells treated with 0 μM, 0.2 μM, 0.4 μM TAE684 and migrated across the membrane were stained with crystal violet and imaged at 100× magnification (*n* = 3). **D**–**F** SK-MEL-28 cells were injected into nude mice to establish subcutaneous xenografts. Once tumors were palpable, the mice were randomized for oral gavage of PEG300 (vehicle control), 5 or 10 mg/kg TAE684 daily. Tumor volumes (**E**) and body weights (**F**) were measured every 2 days (*n* = 6). **G** Immunohistochemistry staining of Ki67 (1:400) in xenografted melanoma mouse model tissues as described in section Materials and Methods. Bar chart graphs of Ki67 positive rate (%) were shown (*n* = 3). The significance of differences was evaluated using one-way ANOVA. *p < 0.05; **p < 0.01; ***p  < 0.001; ****p < 0.0001.

Melanoma is characterized by its highly aggressive nature. Consequently, we detected the impacts of TAE684 on the clonogenicity, migration and invasion ability of melanoma cells via wound healing assays and transwell assays. TAE684 significantly inhibited the clonogenicity of SK-MEL-28, A375 and SK-MEL-5 (Fig. 2A, Fig. S3B). In addition, TAE684 impeded both cell migration and invasion in a concentrate-related manner (Fig. 2B, C, Fig. S3C, D). To evaluate the in vivo anti-cancer efficacy of TAE684, SK-MEL-28 subcutaneous tumor formation followed by TAE684 daily gavage was conducted in BALB/c-nu mice. We found that TAE684 suppressed melanoma growth with no apparent toxicity observed over the 20 day treatment period (Fig. 2D–F). Immunohistochemistry revealed a reduced Ki67 positive ratio in the TAE684 treatment group (Fig. 2G, Fig. S3E). In summary, TAE684 effectively inhibits the malignant phenotypes of melanoma.

### TAE684 induces apoptosis by causing ROS accumulation, DNA damage, and G2/M cell cycle arrest

**Fig. 3:**
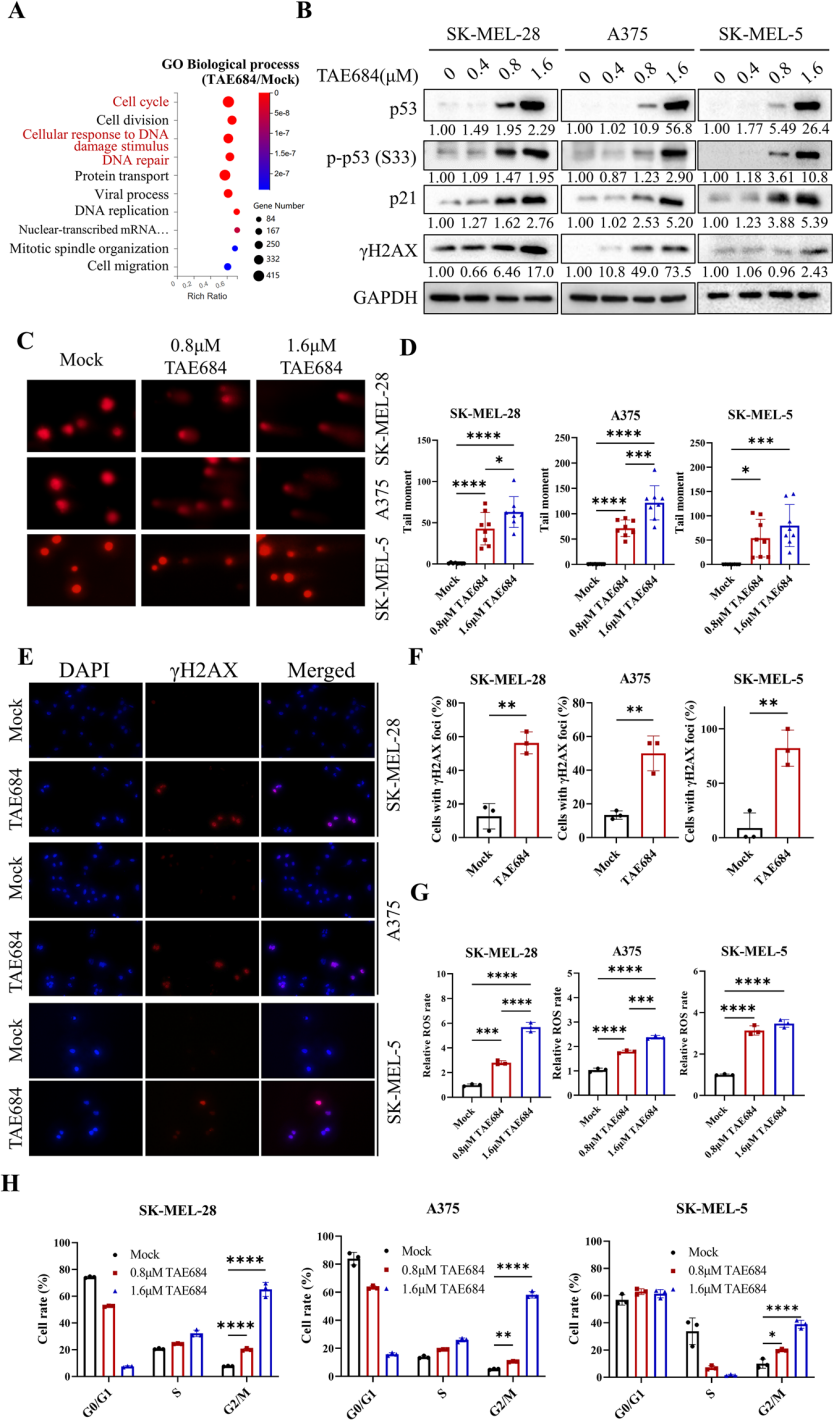
TAE684 induces apoptosis by causing DNA damage and cell cycle arrest in melanoma cells. **A** Whole transcriptome sequencing of SK-MEL-28 cells before and after 48 h of 0.8 μM TAE684 treatment was done as described in the section Materials and Methods. Gene enrichment analysis showing the top enriched GO biological processes. **B** After treating with a series of concentrations of TAE684 for 48 h, SK-MEL-28, A375 and SK-MEL-5 cells were collected for immunoblotting. Indicator proteins of DNA damage and cell cycle arrest were detected by specific antibodies and GAPDH was used as an internal control. **C**, **D** Melanoma cells were treated with TAE684 for 48 h and subjected to an alkaline comet assay. Images were captured by a fluorescence microscope and analyzed by CASP software (*n* = 8). **E**, **F** γH2AX in melanoma cells was stained by immunofluorescence and counted after the cells were treated with 0.8 μM TAE684 for 48 h (*n* = 3). **G** Flow cytometric analysis of relative DCFH-DA fluorescence was performed to measure ROS levels in melanoma cells treated with TAE684 (0.8 μM or 1.6 μM) for 6 h (*n* = 3). **H** Cell cycle distribution was detected by flow cytometry in melanoma cells treated with TAE684 (0.8 μM or 1.6 μM) for 48 h according to the manufacturer’s instruction (*n* = 3). The significance of differences was evaluated using one-way ANOVA (**D**−**H**) and Student’s *t*-test (**F**). *p < 0.05; **p < 0.01; ***p < 0.001; ****p < 0.0001.

Previous studies have indicated that Fyn inhibition leads to apoptosis, although the specific upstream mechanisms remain unclear [24]. Flow cytometric analysis revealed that 48 h of TAE684 treatment upregulated the ratio of apoptotic melanoma cells (Fig. S4A). Meanwhile, elevated BAX and cleaved PARP, along with decreased Bcl2 was detected in TAE684-treated cells (Fig. S4B). RNA-seq analysis revealed significant alterations in DNA damage-related pathway after TAE684 treatment for 48 h at a concentration of 0.8 μM (Fig. 3A). In all three melanoma cell lines, TAE684 elevated the expression of p53, phosho-p53(p-p53), p21, and γH2AX (Fig. 3B). Comet assays demonstrated that longer tail moments were induced by TAE684 in melanoma cells, which indicated severer DNA damage level (Fig. 3C, D). Through immunofluorescence assay, we assessed the formation of γH2AX foci, a sign of DNA double-strand breaks, and noted a rise in γH2AX positive cells post TAE684 treatment. (Fig. 3E–F). Given that elevated intracellular ROS levels can lead to DNA damage [35], we investigated ROS levels before and after TAE684 treatment. Flow cytometry analysis revealed higher ROS levels in melanoma cells post TAE684 incubation (Fig. 3G). Cell cycle analysis indicated that TAE684 induced notable G2/M phase arrest depending on the concentration (Fig. 3H, Fig. S4C). To determine whether the ROS elevation might also trigger alternative forms of cell death, melanoma cells were co-treated with TAE684 and a panel of cell death inhibitors, including Z-VAD-fmk, Ferrostatin-1, Necrostatin-1, and Chloroquine. Among these, only Z-VAD-fmk partially rescued cell viability, indicating that apoptosis is the main form of TAE684-induced cell death (Fig. S5). Notably, the reduced viability was primarily due to G2/M cell cycle arrest rather than extensive cell death. In summary, TAE684 increases ROS levels to induce DNA damage, G2/M arrest, and apoptosis, thereby inhibiting melanoma progression.

### Fyn inhibition causes DNA damage and G2/M cell cycle arrest

**Fig. 4:**
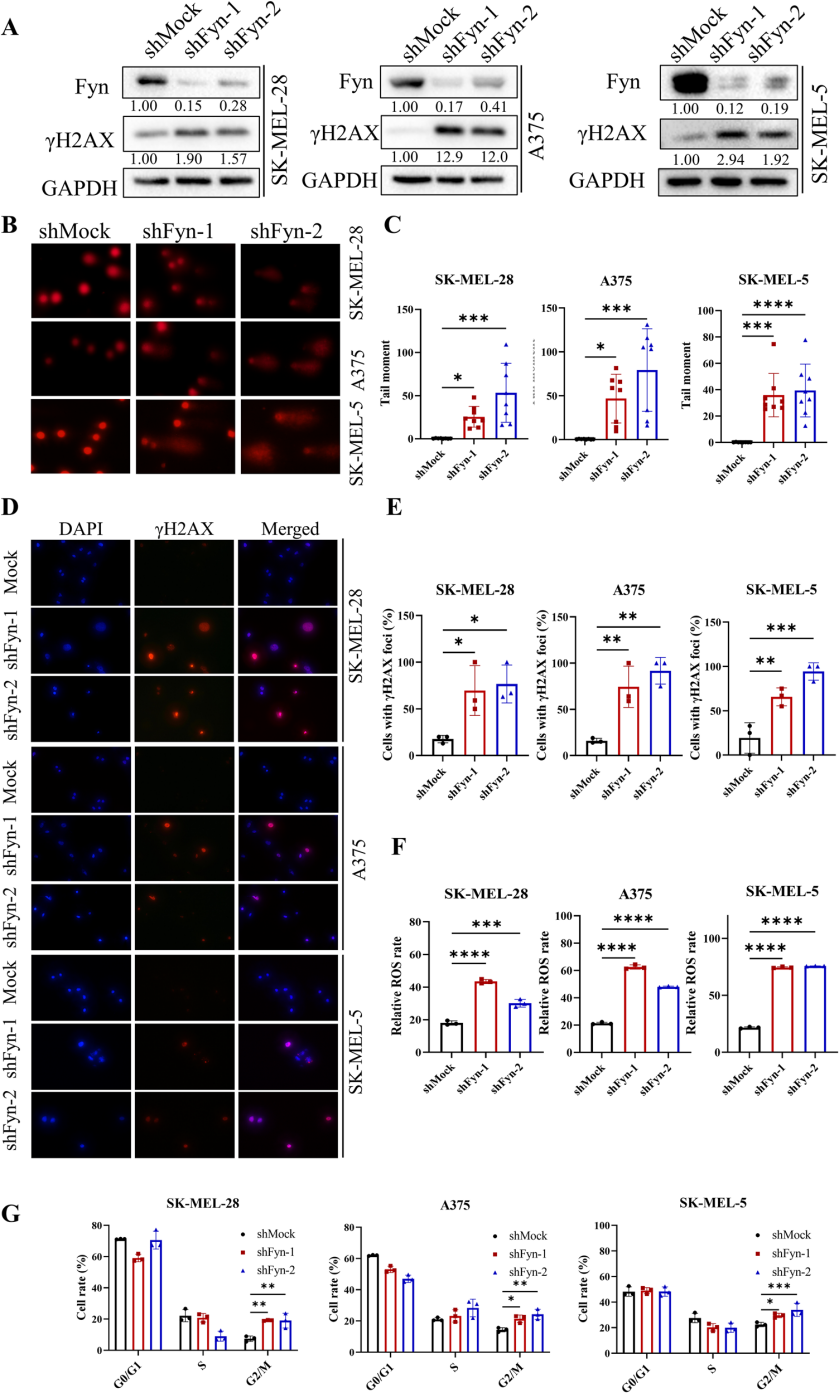
Knockdown of Fyn induces apoptosis by causing DNA damage and cell cycle arrest in melanoma cells. **A** Melanoma cells after the knockdown of Fyn were collected for immunoblotting. Indicated proteins were detected by specific antibodies and GAPDH was used as an internal control. **B**, **C** Melanoma cells after knockdown of Fyn were subjected to alkaline comet assay. Images were captured by a fluorescence microscope and analyzed by CASP software, as described in materials and methods (*n* = 6). **D**, **E** γH2AX in melanoma cells was stained by immunofluorescence and counted after the knockdown of Fyn (*n* = 3). **F** Flow cytometric analysis of relative DCFH-DA fluorescence was performed to measure ROS levels in Melanoma Fyn-knockdown cells (*n* = 3). **G** Cell cycle distribution was detected by flow cytometry in Fyn-knockdown melanoma cells according to the manufacturer’s instructions (*n* = 3). The significance of differences was evaluated using one-way ANOVA. *p  < 0.05; **p  < 0.01; ***p  < 0.001; ****p  < 0.0001.

We generated Fyn-knockdown SK-MEL-28, A375 and SK-MEL-5 melanoma cells through lentivirus infection. Herein, we found that Fyn knockdown elevated γH2AX (Fig. 4A). Comet assays revealed that all three melanoma cell lines displayed longer tail moments after knockdown of Fyn (Fig. 4B, C), as well as a more γH2AX positive cells in immunofluorescence assay (Fig. 4D, E). Increased level of ROS and apparent G2/M cell cycle arrest were observed in Fyn-knockdown melanoma cells, consistent with the results in TAE684-treated samples (Fig. 4F, G, Fig. S6). Therefore, Fyn inhibition leads to melanoma apoptosis through induce high levels of ROS followed by DNA damage.

### Targeting Fyn leads to the inhibition of the AP-1 signaling pathway

**Fig. 5:**
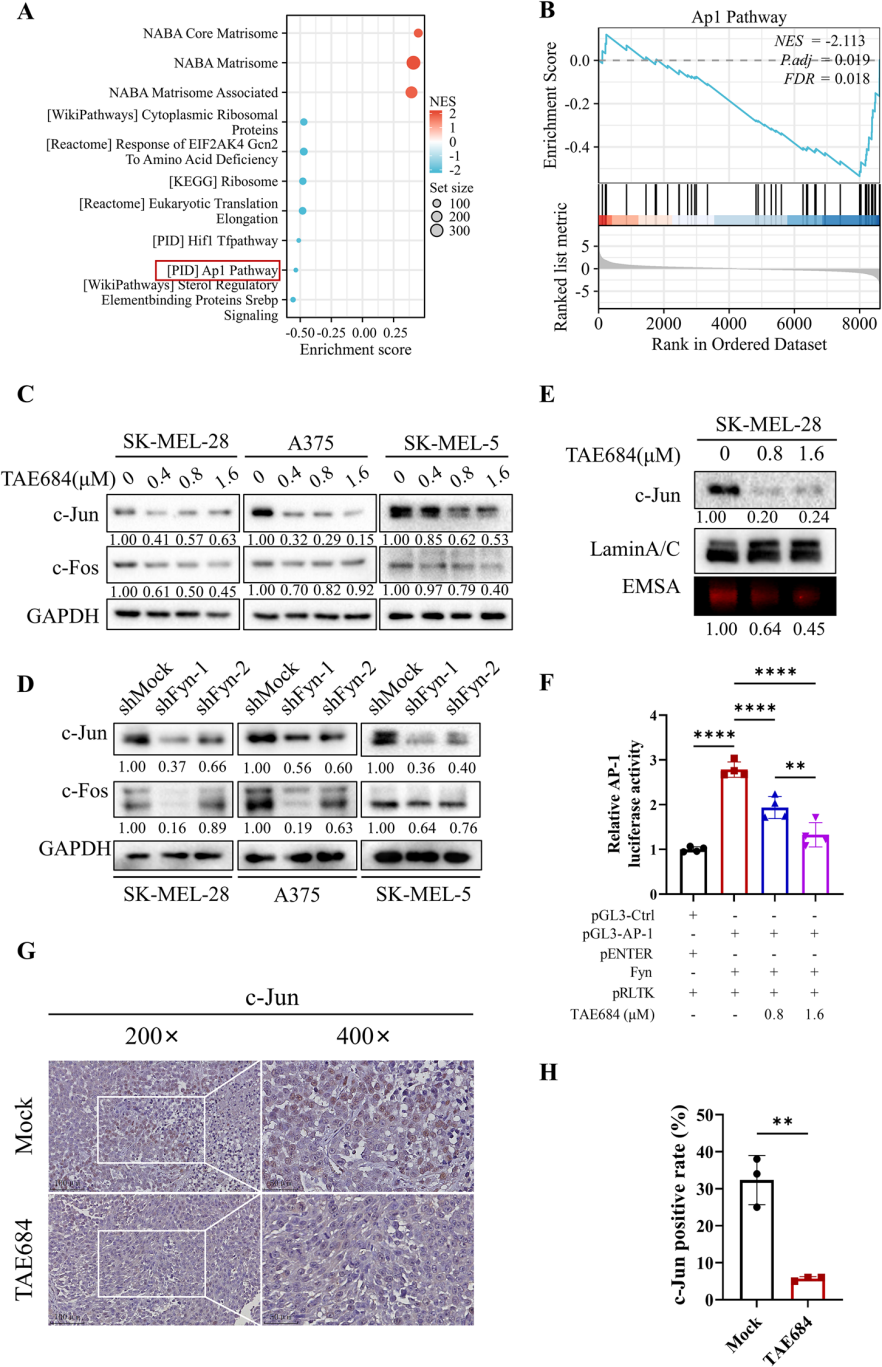
Targeting Fyn results in inhibition of the AP-1 signaling pathway. **A**, **B** GSEA and visualization were performed using ggplot2[3.4.4] (**C**-**D**). Melanoma cells after being treated with a series of concentrations of TAE684 for 48 h (**C**) or knockdown of Fyn (**D**) were collected for immunoblotting. C-Jun and c-Fos were detected by specific antibodies and GAPDH was used as an internal control. **E** Nuclear protein was extracted from TAE684-treated melanoma cells and subjected to immunoblot analysis using antibodies to c-Jun or electrophoretic mobility-shift assays. Lamin A/C served as an internal control. **F** AP-1 luciferase activity is regulated by Fyn in HEK293T cells. Dual-luciferase reporter assay performed by co-transfection of luciferase reporter pGL3-AP-1-luc and Renilla luciferase plasmid with Fyn plasmid or negative control in HEK293T cells (*n* = 3). After 24 h of transfection, the medium was replaced with DMEM containing DMSO, 0.8 μM or 1.6 μM TAE684. Luciferase activity was determined 48 h after co-transfection. **G**, **H**. Immunohistochemistry staining of c-Jun (1:600) in xenografted melanoma mouse model tissues as described previously. Representative images were taken (**G**) and bar chart graphs of the c-Jun positive rate (%) were shown (**H**) (*n* = 3). The significance of differences was evaluated using one-way ANOVA (**F**) and Student’s *t*-test (**H**). **p < 0.01; ****p < 0.0001.

To gain a clearer insight into the impact of TAE684 on melanoma cells, GSEA was conducted based on the RNA-seq data. SK-MEL-28 treated with 0.8 μM TAE684 for 48 h revealed a down-regulation of AP-1 signaling pathway (Fig. 5A, B). Since c-Jun and c-Fos are core components of the AP-1 transcription factor and play key roles in the activation of the AP-1 signaling pathway, we assessed their expression to evaluate the impact of TAE684 [36]. Upon treating SK-MEL-28, A375 and SK-MEL-5 cells with different concentrations of TAE684, we observed down-regulation of c-Fos and c-Jun at both transcriptional and translational levels (Fig. 5C, Fig. S7A). Similar changes were noted in the Fyn-knockdown cells (Fig. 5D, Fig. S7B). As transcription factors, AP-1 family members translocate to the nucleus to facilitate the expression of target genes [37]. Since c-Jun was more obviously down-regulated, we further investigate its role in transcription. We found that TAE684 treatment led to lower intranuclear c-Jun level as confirmed by EMSA (Fig. 5E). We also performed a dual-luciferase reporter assay in 293 T cells using a luciferase plasmid driven by the AP-1 promoter and found that Fyn overexpression upregulated AP-1 promoter activity, while TAE684 inhibited Fyn and subsequently downregulated AP-1 luciferase activity. Furthermore, we confirmed a concentration-dependent decrease of c-Jun positive cell rate (%) within TAE684 treated tumors via immunohistochemistry (Fig. 5G, H).

### TAE684 inhibits the malignant traits of vemurafenib-resistant melanoma cells by mediating DNA damage

**Fig. 6:**
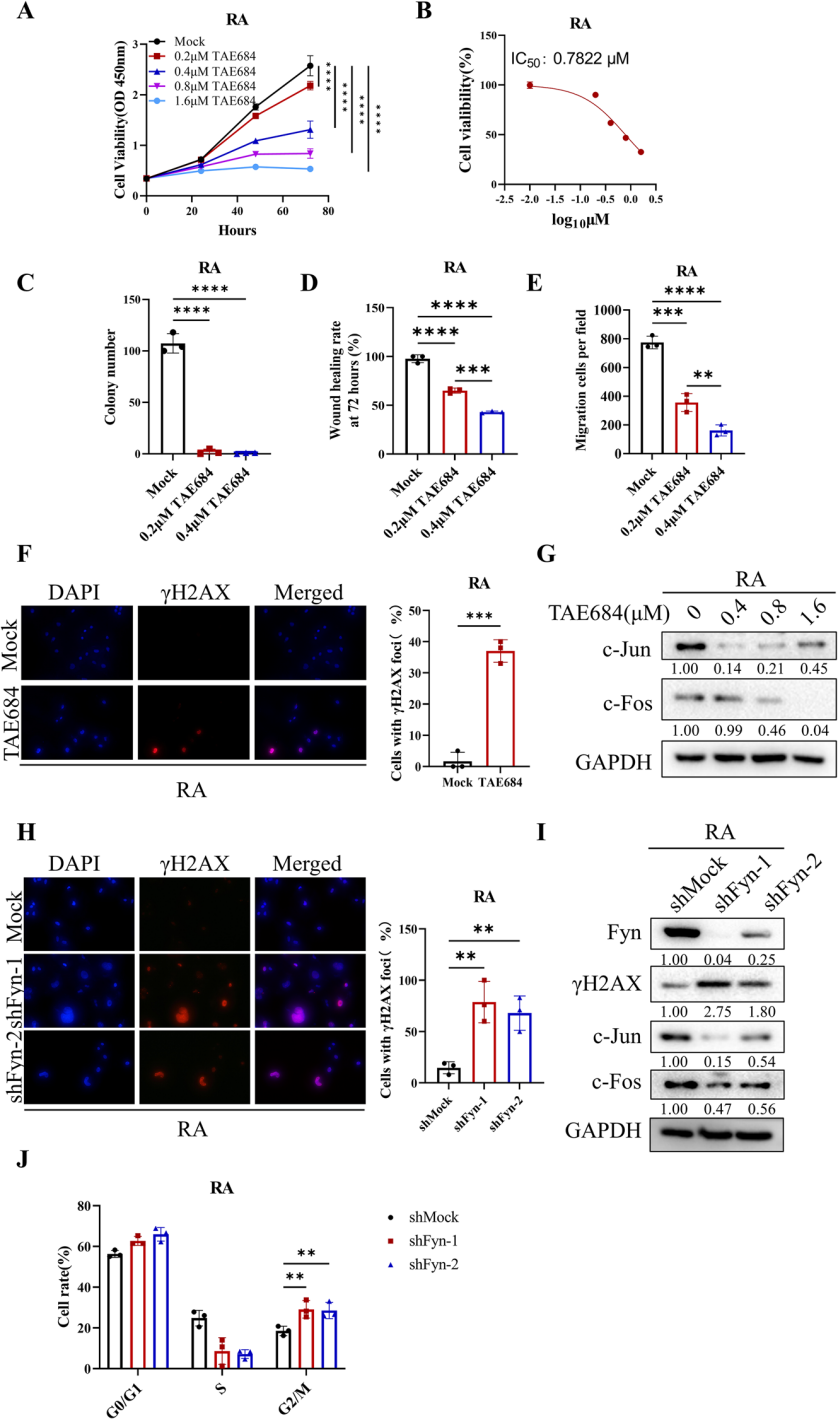
TAE684 inhibits the malignant phenotype of vemurafenib-resistant melanoma cells by mediating DNA damage and down-regulating the AP-1 signaling pathway. **A**, **B** CCK-8 OD values at 450 nm of RA cells treated with 0 μM, 0.2 μM, 0.4 μM, 0.8 μM, 1.6 μM TAE684 at 0 h, 24 h, 48 h, and 72 h. The value of IC_50_ was calculated by GraphPad Prism 9 (*n* = 6). **C** Colony formation assay to detect the number of RA cell clones at different concentrations (0 μM, 0.2 μM, 0.4 μM) of TAE684. The clones were stained with crystal violet and counted with image J software (*n* = 3). **D** Wound healing assays were performed to assess the invasion ability of RA treated with different concentrations (0, 0.2 µM, 0.4 µM) of TAE684. The cell wound area at 72 h was analyzed using ImageJ software, and the wound healing rate was statistically analyzed (*n* = 3). **E** In transwell assays, RA treated with TAE684 and migrated across the membrane were stained with crystal violet and imaged at 100× magnification (*n* = 3). **F**, **H** RA cells after being treated with a series of concentrations of TAE684 for 48 h (**F**) or knockdown of Fyn (**H**) were collected for immunoblotting. C-Jun and c-Fos were detected by specific antibodies and GAPDH was used as an internal control. **G**, **I** γH2AX in melanoma cells was stained by immunofluorescence after the cells were treated with 0.8 μM TAE684 for 48 h (**G**) or knockdown of Fyn (**I**). **J** γH2AX positive rate were presented as the mean (*n* = 3) ± SD. **K** Cell cycle distribution was detected by flow cytometry in RA Fyn-knockdown cells according to the manufacturer’s instructions (*n* = 3). The significance of differences was evaluated using one-way ANOVA. **p < 0.01; ***p < 0.001; ****p < 0.0001.

Given the reported role of Fyn in drug resistance, we explored whether TAE684 could overcome vemurafenib resistance in melanoma by targeting Fyn. To this end, we applied TAE684 to vemurafenib-resistant A375 (BRAF V600E) cells (referred to as RA). TAE684 significantly impacted the proliferation, clonogenicity, migration, and invasion capabilities of RA cells, with an IC_50_ value of 0.7822 μM, which is close to that of parental A375 cells (Fig. 6A–E, Fig. S7A–C). This result demonstrated that vemurafenib-resistant melanoma cells did not develop cross-resistance to TAE684. Additionally, TAE684-induced DNA damage was confirmed by immunofluorescence, and downregulation of the AP-1 signaling pathway at both mRNA and protein levels was demonstrated by qPCR and immunoblotting (Fig. 6F, G, Fig. S8D). Similar results were observed in RA after Fyn knockdown, along with G2/M cell cycle arrest (Fig. 6H–J, Fig. S8E, F). These findings indicate that TAE684 could exhibit effective inhibitory function even in BRAFi resistant melanoma cells.

### Targeting Fyn reverses vemurafenib resistance by inhibiting the AP-1 signaling pathway

**Fig. 7:**
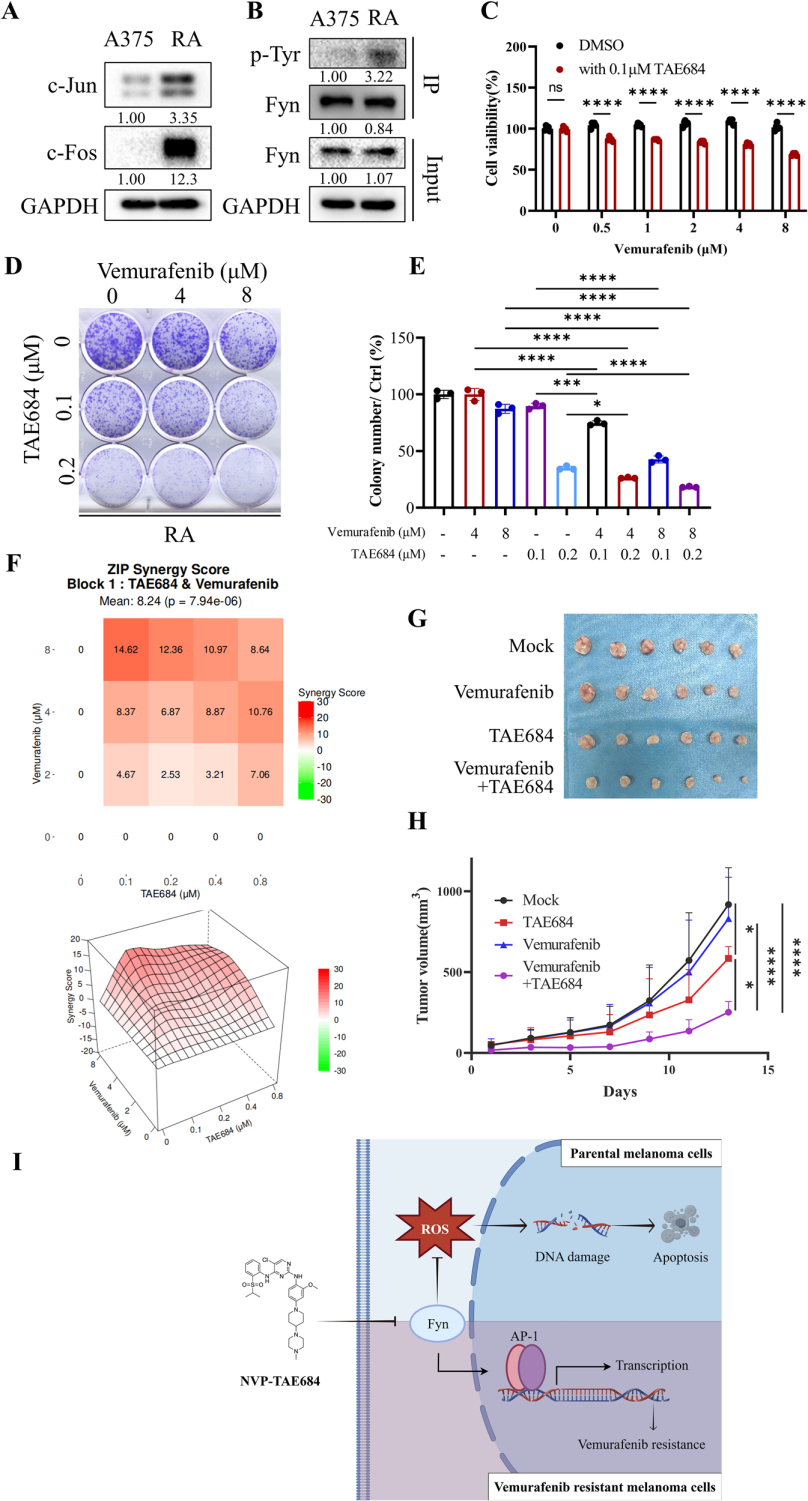
Combination of TAE684 and vemurafenib is an effective strategy for vemurafenib-resistant melanoma. **A** A375 and RA cells were collected for immunoblotting. C-Jun and c-Fos were detected by specific antibodies and GAPDH was used as an internal control (*n* = 5). **B** Immunoprecipitation demonstrated tyrosine phosphorylation levels of Fyn in A375 and RA cells. **C** RA cells were seeded into 96-well plates and treated with 0.1 μM TAE684 combined with indicated concentrations of vemurafenib for 48 h, and cell viability was tested by CCK-8 kit. **D**, **E** Colony formation assay to detect the number of RA cell clones at combinations of different concentrations of TAE684 or vemurafenib. The clones were stained with crystal violet and counted with image J software (*n* = 3). **F** Synergy between TAE684 and vemurafenib in RA cells was analyzed using the SynergyFinder application. The upper panel details the ZIP scores for different concentration combinations, while the lower panel visualizes the average synergy score of the two drugs (8.24, ZIP synergy score). **G**, **H** RA cells were injected into nude mice to establish subcutaneous xenografts. Once tumors were palpable, the mice were randomized for oral gavage of 5 mg/kg TAE684, 10 mg/kg vemurafenib, or 5 mg/kg TAE684 + 10 mg/kg vemurafenib each day. Tumor volumes (**H**) were measured every 2 days (*n* = 6). **I** The mechanism by which TAE684 inhibits the malignant phenotype of parental melanoma cells and reverses the response of drug-resistant melanoma cells to vemurafenib. The schematic diagram was drawn with FigDraw. The significance of differences was evaluated using Student’s *t*-test (**C**) and one-way ANOVA (**E**, **H**). *p  < 0.05; ***p < 0.001; ****p < 0.0001.

Most BRAFi-resistant melanomas reactivate the MAPK pathway through specific mutations, with the AP-1 signaling pathway being one of its downstream components [38]. We confirmed that c-Jun and c-Fos levels were elevated in RA cells compared to the parental A375 cells by immunoblotting (Fig. 7A), which indicated a potential mechanism for vemurafenib acquired resistance. Although Fyn expression did not show significant up-regulation in RA, we found that its tyrosine phosphorylation level was upregulated by immunoprecipitation, implying a higher activation level in drug-resistant cells (Fig. 7B). Co-treatment of RA cells with 0.1 μM TAE684 and different concentrations of vemurafenib significantly reduced cell viability compared to treatment with vemurafenib alone (Fig. 7C). Besides, the combination of the two drugs maximally inhibited clonogenicity (Fig. 7D–E). The effects of combining TAE684 and vemurafenib in RA cells were assessed by SynergyFinder and Compusyn, which exhibited significant synergy effect (Fig. 7F, upper panel; Fig. S9A). Meanwhile, we observed that the combination of 0.1 μM TAE684 and 8 μM vemurafenib achieved the highest synergy score (Fig. 7F, lower panel), indicating that TAE684 at lower concentrations can restore the sensitivity of resistant cells to BRAF inhibitors. Notably, even in treatment-naïve melanoma cells, TAE684 co-treatment enhanced the efficacy of vemurafenib (Fig. S9B). In xenograft model generated by RA cells, tumors in mice treated with both TAE684 and vemurafenib were significantly smaller in size compared to mice treated with either drug alone, with no effect on body weight was observed (Fig. 7G, H, Fig. S9C, D).

To explore the function of c-Jun in acquired resistance, we generated c-Jun overexpression cell lines in Melanoma cells by lentivirus (Fig. S9E). Those cells acquired vemurafenib resistance, further confirming the critical role of the AP-1 signaling pathway in acquired BRAFi resistance (Fig. S9F). In conclusion, we discovered that TAE684 reverses vemurafenib resistance by targeting Fyn and downregulating the AP-1 pathway.

## Discussion

Fyn, a tyrosine kinase, regulates cell growth, survival, adhesion, and motility by phosphorylating multiple substrates, thereby playing a critical role in the development of several cancers [23, 39, 40]. Prior investigations have reported that Fyn is highly expressed in melanoma compared to normal skin tissues. Moreover, Fyn-knockdown significantly suppresses melanoma growth and induces apoptosis [24]. These results emphasize the potential value of Fyn as a therapeutic target for melanoma. To date, several Fyn inhibitors, including saracatinib, dasatinib, ponatinib, bosutinib, and repotrectinib, have undergone clinical trials [41]. Saracatinib exhibits significant activity against Fyn kinase, with an IC_50_ of 10 nM [42]. However, in phase II clinical trials, it showed limited efficacy in patients with advanced melanoma, metastatic colorectal cancer, or malignant thymic tumors. Additionally, potential immunosuppressive effects of saracatinib were reported [43–45]. Dasatinib, an FDA-approved drug for chronic granulocytic leukemia and gastrointestinal mesenchymal stromal tumors, demonstrated limited efficacy and significant adverse reactions in a clinical trial involving advanced melanoma [46]. Ponatinib, bosutinib, and repotrectinib are FDA-approved for the treatment of acute lymphoblastic leukemia, childhood chronic myeloid leukemia, and ROS1-positive non-small cell lung cancer, respectively. However, their efficacy in melanoma is still not fully understood [47–49]. In search of novel Fyn-targeted inhibitors against melanoma, we identified a small molecule compound TAE684 by virtual screening and confirmed its binding and effects through in vitro and in vivo experiments. TAE684 was initially discovered as an ALK inhibitor for tumors with ALK mutations with significant efficacy [34, 50]. We found that TAE684 induced apoptosis in melanoma cell lines, which was consistent with the phenotype in Fyn-knockdown cells and involved a mechanism independent of its original target ALK. These results raise the potential of TAE684 as a Fyn inhibitor for clinical treatment of melanoma, although its efficacy and safety need to be further evaluated.

We previously reported that Fyn inhibition can cause apoptosis, although the upstream mechanisms need to be further elucidated [24]. In this study, we found that Fyn inhibition-induced apoptosis was associated with ROS accumulation, DNA damage, and G2/M cell cycle arrest. ROS consist of a series of oxygen-containing free radicals generated during mitochondrial metabolism (O2-, H2, O2, and -OH, among others), and their excessive accumulation leads to cellular damage [35]. The Nrf2-mediated antioxidant system is an important pathway for the scavenging of ROS [51], while Fyn can regulate Nrf2 activity through the erythropoietin signaling pathway, which is important for maintaining ROS homeostasis [52]. Methylmercury increase ROS levels through down-regulation Fyn in astrocytes and does harm to nervous system [53]. Fucoxanthin and rosemarinic acid are able to prevent Alzheimer’s disease by stimulating the Nrf2-mediated antioxidant system through Akt/GSK-3β/Fyn pathway [37, 54]. We verified for the first time in melanoma that knockdown of Fyn has an up-regulatory effect on intracellular ROS levels. ROS directly activate TP53, disrupt the homeostasis between members of the Bcl2 family of proteins, and induce caspase-dependent apoptosis [55, 56]. At the same time, ROS are also a well-known mediator of DNA damage, resulting in DNA strand breaks, base modifications, alkaline sites, and cross-linking of DNA proteins [57]. After DNA damage, the cell cycle enters a state of arrest for DNA repair; however, in the absence of repair, apoptosis is triggered in order to prevent the spread of the genetic lesion to progeny cells [58, 59]. Thus, knockdown of Fyn or TAE684 treatment in melanoma cells further induces apoptosis by inducing intracellular ROS accumulation, leading to DNA damage and cell cycle arrest. In addition, we found that inhibition of Fyn inhibited the AP-1 signaling pathway. AP-1 regulates the expression of downstream target genes such as sulfiredoxin, REDD1, TREX1, and protects against cellular oxidative stress [60–62]. JunB can mediate the transcriptional activation of catalase genes, which are involved in the scavenging of ROS [63]. C-fos deficient cells are related to sensitivity to DNA damage induced by UV radiation and chemotherapeutic agent [64, 65], suggesting the critical position of the AP-1 signaling pathway in the defense against genotoxic agents. Taken together, Fyn inhibition may be down-regulating the AP-1 pathway, thereby promoting ROS production and DNA damage.

BRAF inhibitors are first-line treatments for melanoma patients with the BRAF V600E mutation. However, almost all patients develop resistance shortly after initial treatment. The mechanisms of resistance are classified into two categories: MEK/ERK-dependent and MEK/ERK-independent pathways. The MEK/ERK-dependent pathways are primarily characterized by secondary activation of molecular switches in the MAPK pathway. In contrast, the MEK/ERK-independent mechanisms involve the inactivation of oncogenes, activation of multiple signaling pathways such as PI3K/AKT and STAT3, metabolic reprogramming, and other molecular alterations [66]. These findings highlight the complex molecular changes in BRAFi-resistant tumors. Although BRAFi is usually used in combination with MEKi in clinical settings, resistance to BRAFi alone plays a central role in the development of resistance to the combination therapy [67, 68]. Therefore, investigating BRAFi monotherapy resistance remains highly relevant for understanding the underlying mechanisms of acquired resistance.

AP-1 signaling pathway is activated by the MAPK cascade and plays a crucial role in tumor resistance to BRAFi [38]. In 2015, Mohammad Fallahi-Sichani et al. first demonstrated that AP-1 signaling is upregulated in the adaptive resistance to vemurafenib [69]. C-Jun, a key component of the AP-1 complex, induces the expression of RHOB and activates the AKT signaling pathway, promoting the survival of melanoma cells under BRAFi stress [70]. Furthermore, c-Jun also upregulates genes involved in cell adhesion, epithelial-mesenchymal transition, and ECM molecules, which activate FAK and downstream Src kinases. This process allows melanoma cells to acquire a unique phenotype independent of ERK signaling, thus conferring resistance to BRAFi [71]. Recent studies have also documented that Fyn enhances AP-1 transcriptional activity, further supporting the role of Fyn in mediating drug resistance [72, 73]. Our finding that TAE684 inhibits the AP-1 signaling pathway in RA by targeting Fyn matches those observations in earlier studies. This provides important insights into how TAE684 can act synergistically with vemurafenib to overcome resistance in melanoma cells.

With this study, we firstly demonstrated that Fyn inhibition facilitates apoptosis through a DNA damage related mechanism. Fyn promotes acquired resistance to vemurafenib in melanoma by modulating the AP-1 signaling pathway. TAE684, as a Fyn inhibitor, suppresses malignant phenotypes of parental cells as well as reverses vemurafenib resistance in melanoma. The combination of TAE684 and vemurafenib provides a strategy for future practice on treating BRAFi-resistant melanoma.

## Supplementary information

Figure S1

Figure S2

Figure S3

Figure S4

Figure S5

Figure S6

Figure S7

Figure S8

Figure S9

Supplementary materials

Full Length Western Blot

## Data Availability

The data supporting this study’s findings are available on request from the corresponding author.
