# Supplementary figures and images for: Fyn inhibition by TAE684: A synergistic strategy to suppress melanoma and reverse vemurafenib resistance
Source: Cell Death Dis. 2025 Nov 6;16(1):796. doi: 10.1038/s41419-025-08090-1 (PMC12592403; doi:10.1038/s41419-025-08090-1)

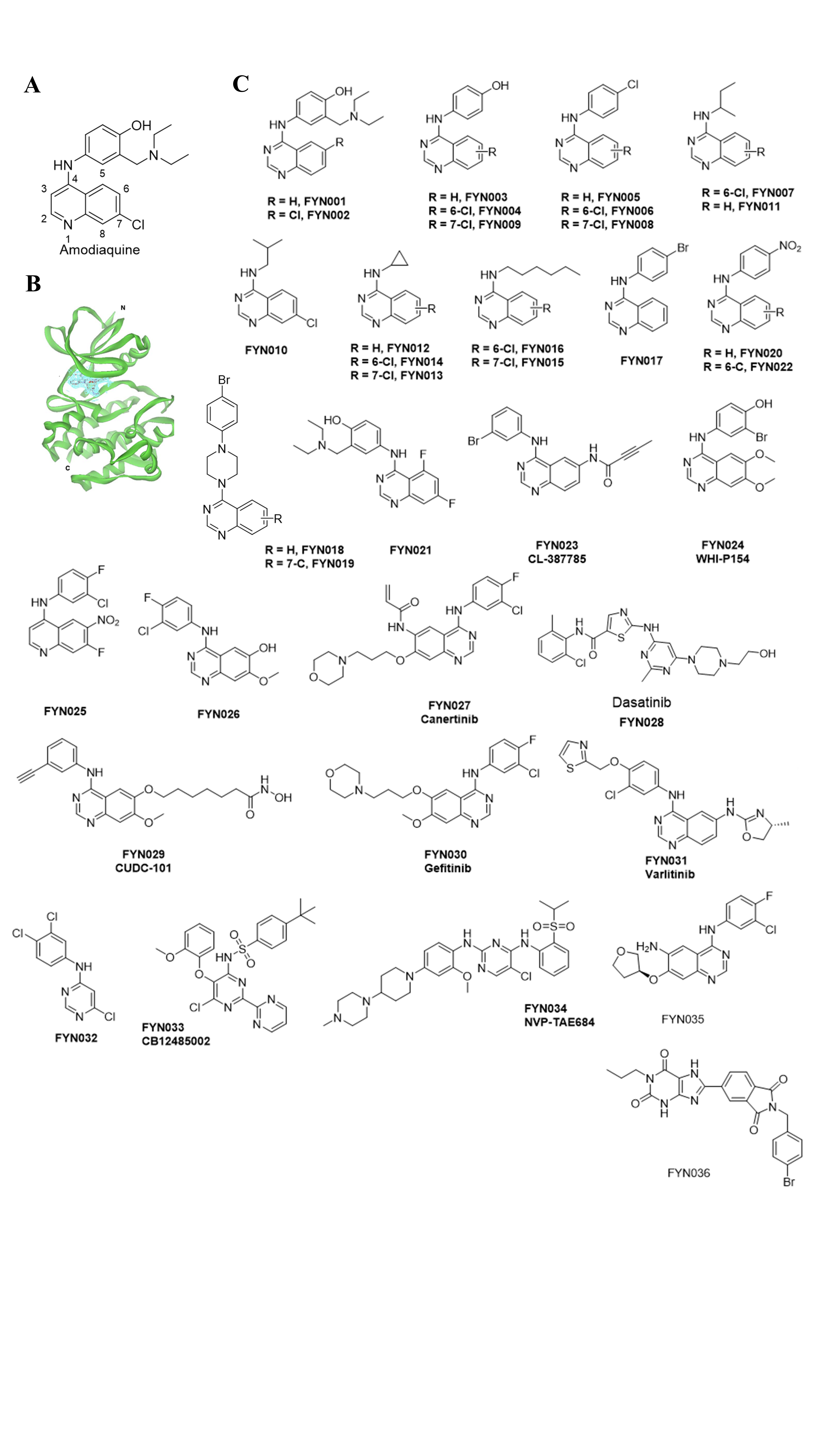

Supplement: Supplementary file 1 — Figure S1 [file 41419_2025_8090_MOESM1_ESM.tif]

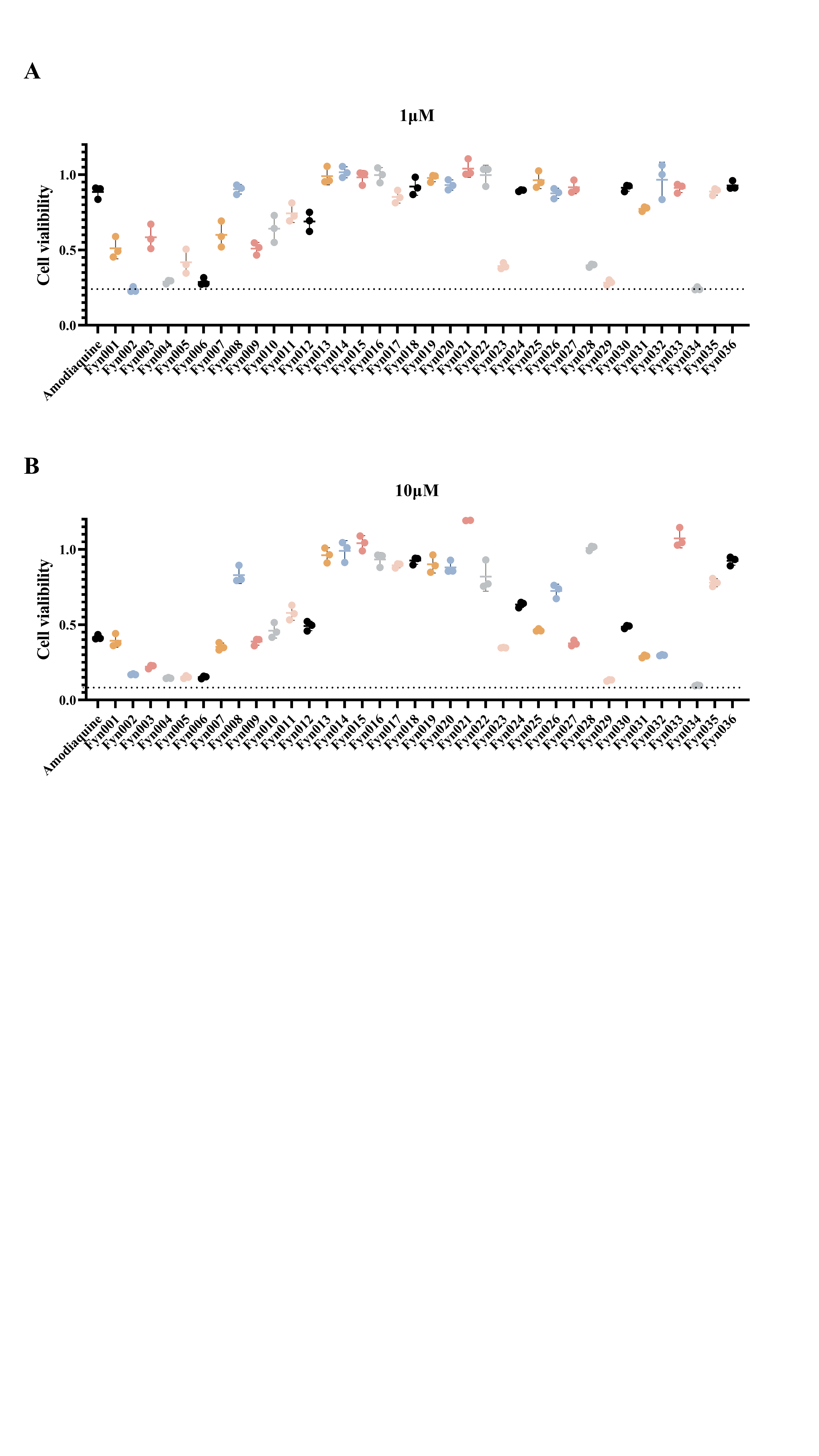

Supplement: Supplementary file 2 — Figure S2 [file 41419_2025_8090_MOESM2_ESM.tif]

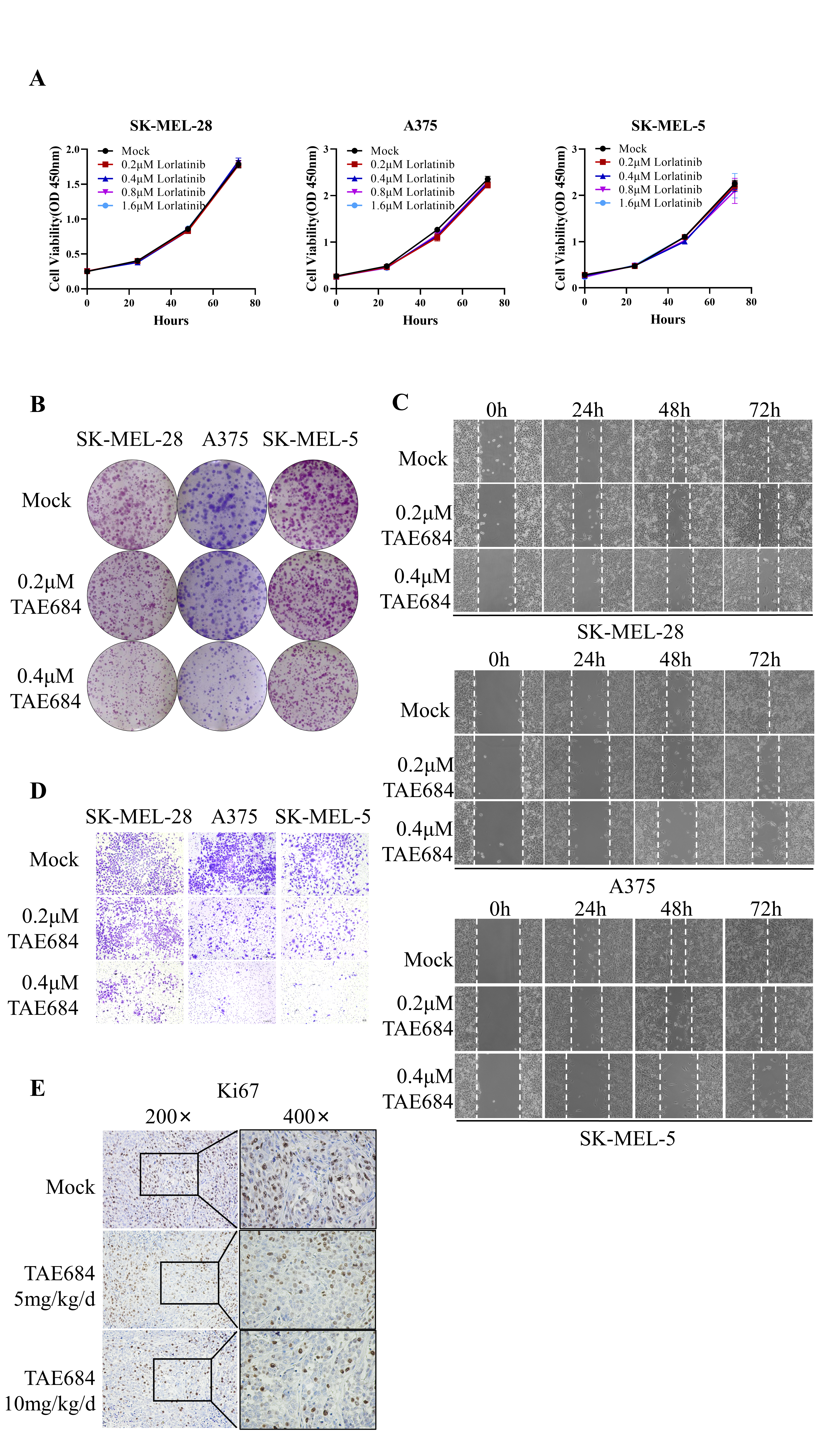

Supplement: Supplementary file 3 — Figure S3 [file 41419_2025_8090_MOESM3_ESM.tif]

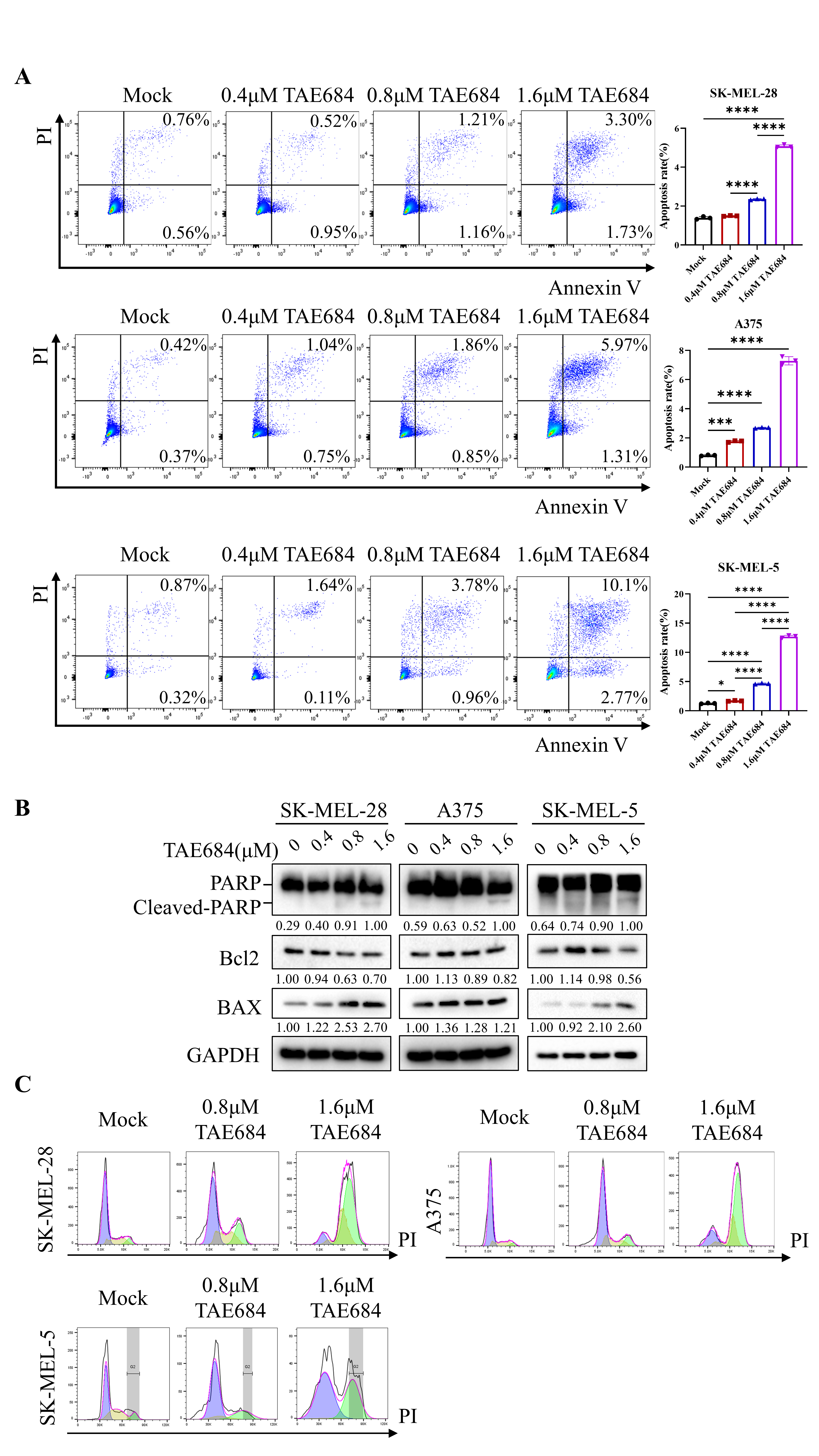

Supplement: Supplementary file 4 — Figure S4 [file 41419_2025_8090_MOESM4_ESM.tif]

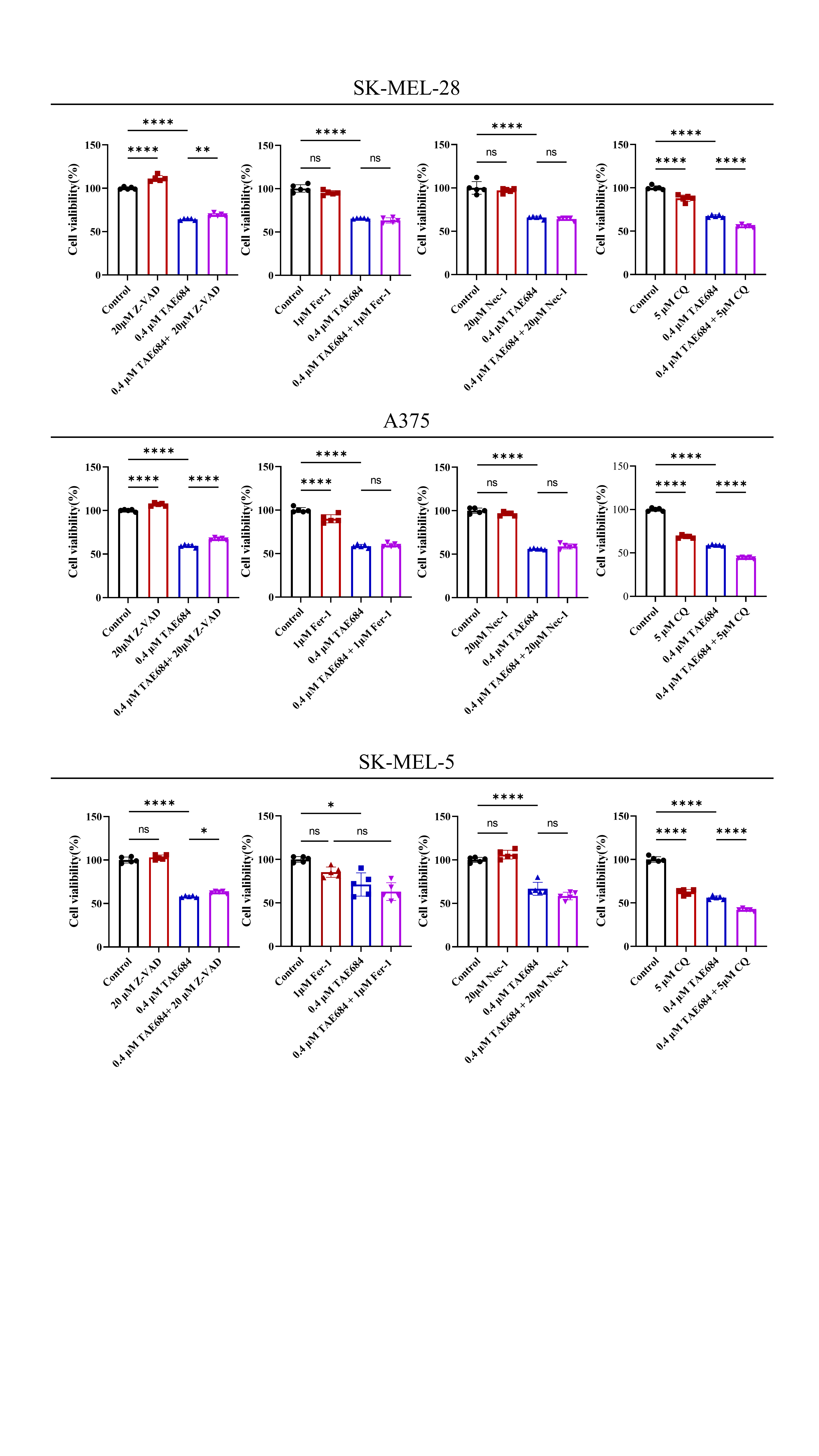

Supplement: Supplementary file 5 — Figure S5 [file 41419_2025_8090_MOESM5_ESM.tif]

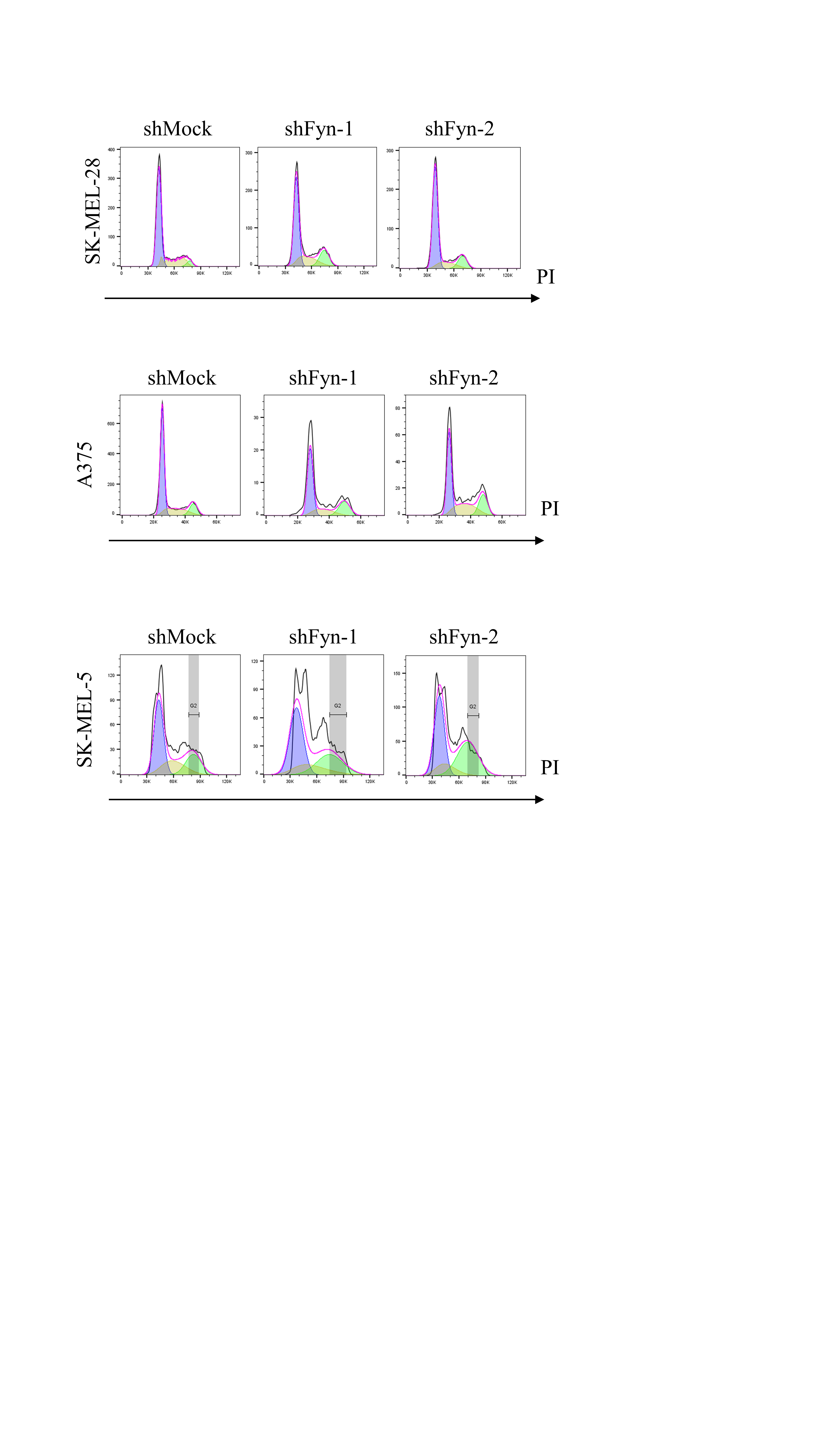

Supplement: Supplementary file 6 — Figure S6 [file 41419_2025_8090_MOESM6_ESM.tif]

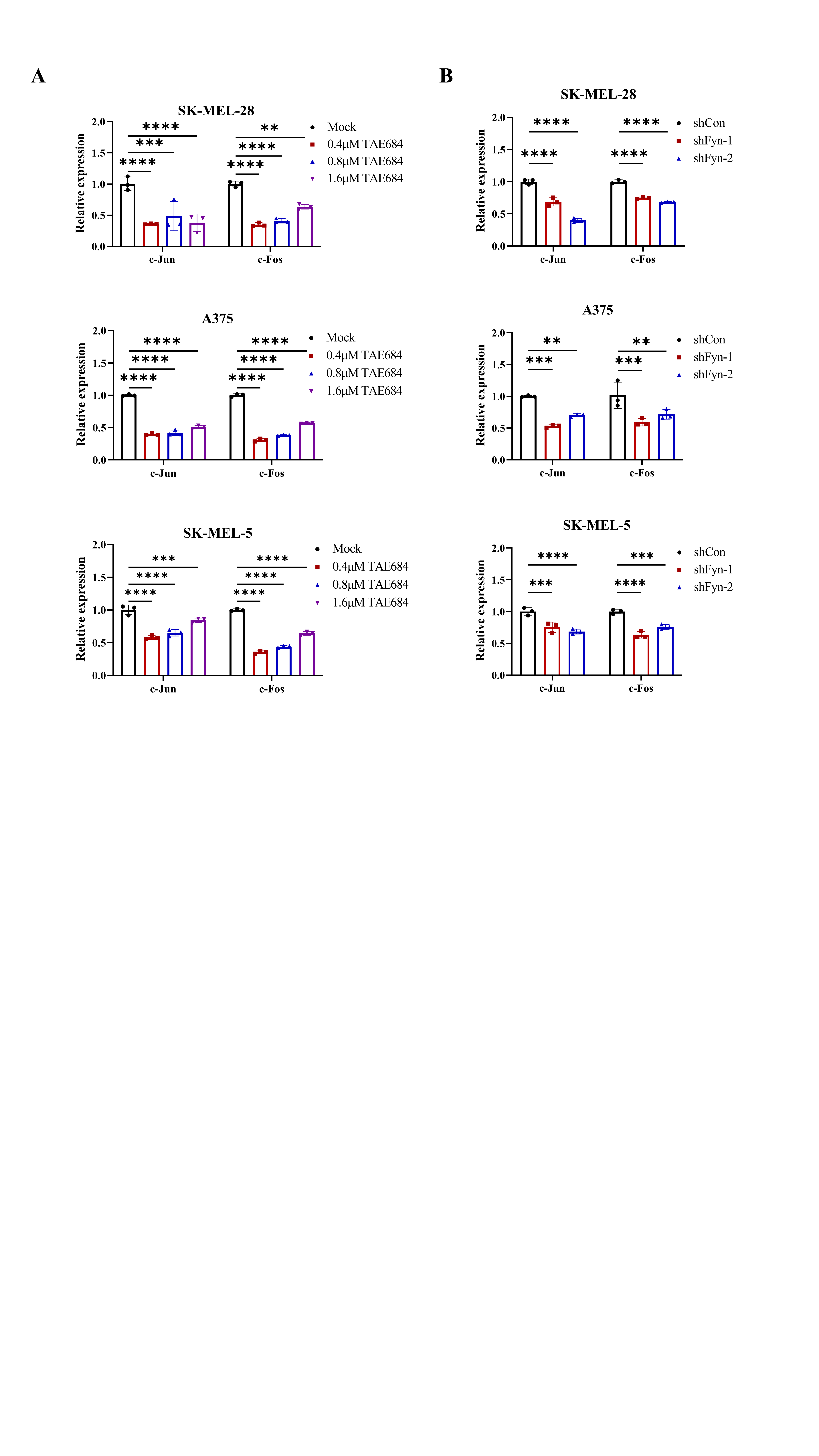

Supplement: Supplementary file 7 — Figure S7 [file 41419_2025_8090_MOESM7_ESM.tif]

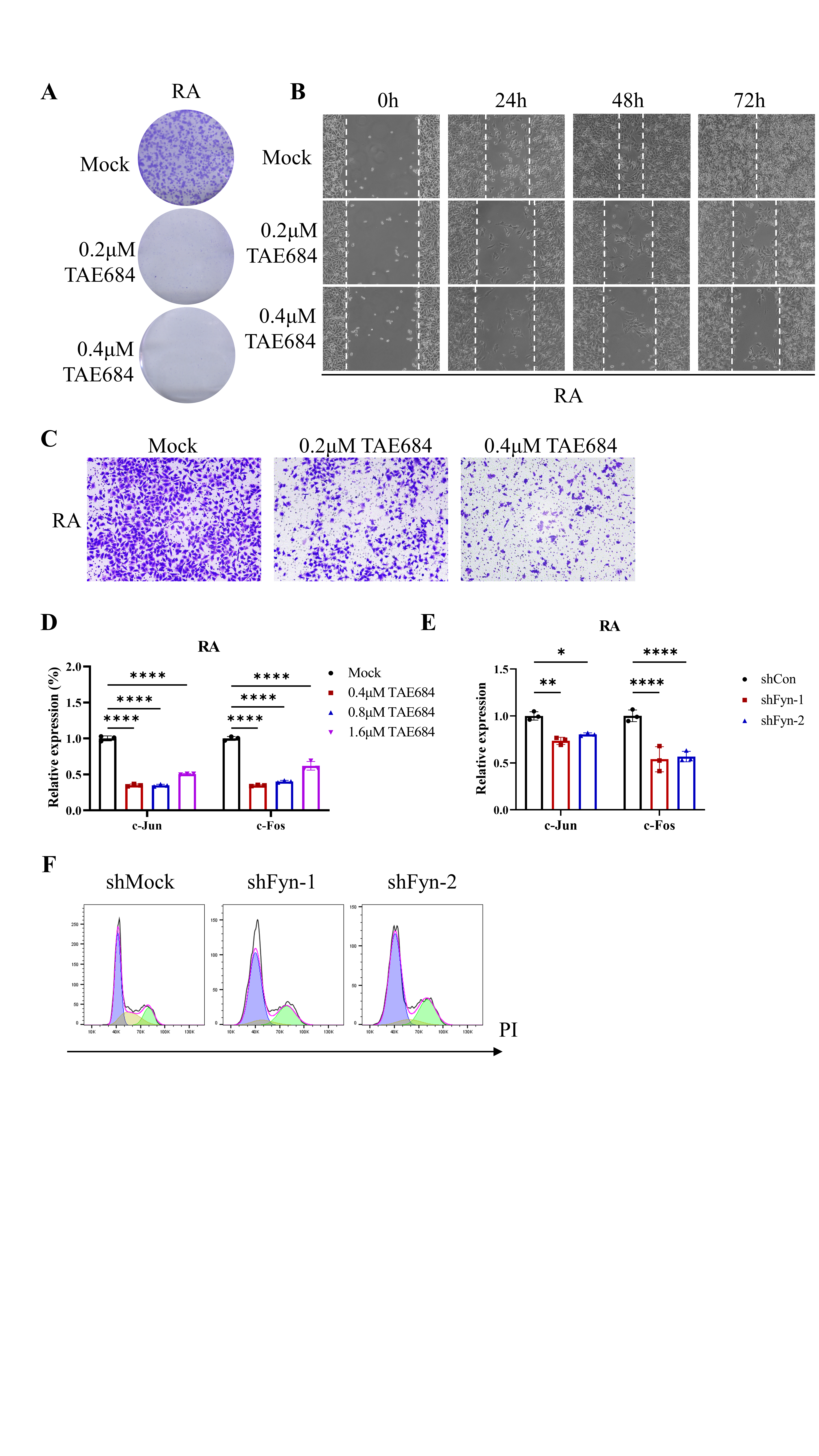

Supplement: Supplementary file 8 — Figure S8 [file 41419_2025_8090_MOESM8_ESM.tif]

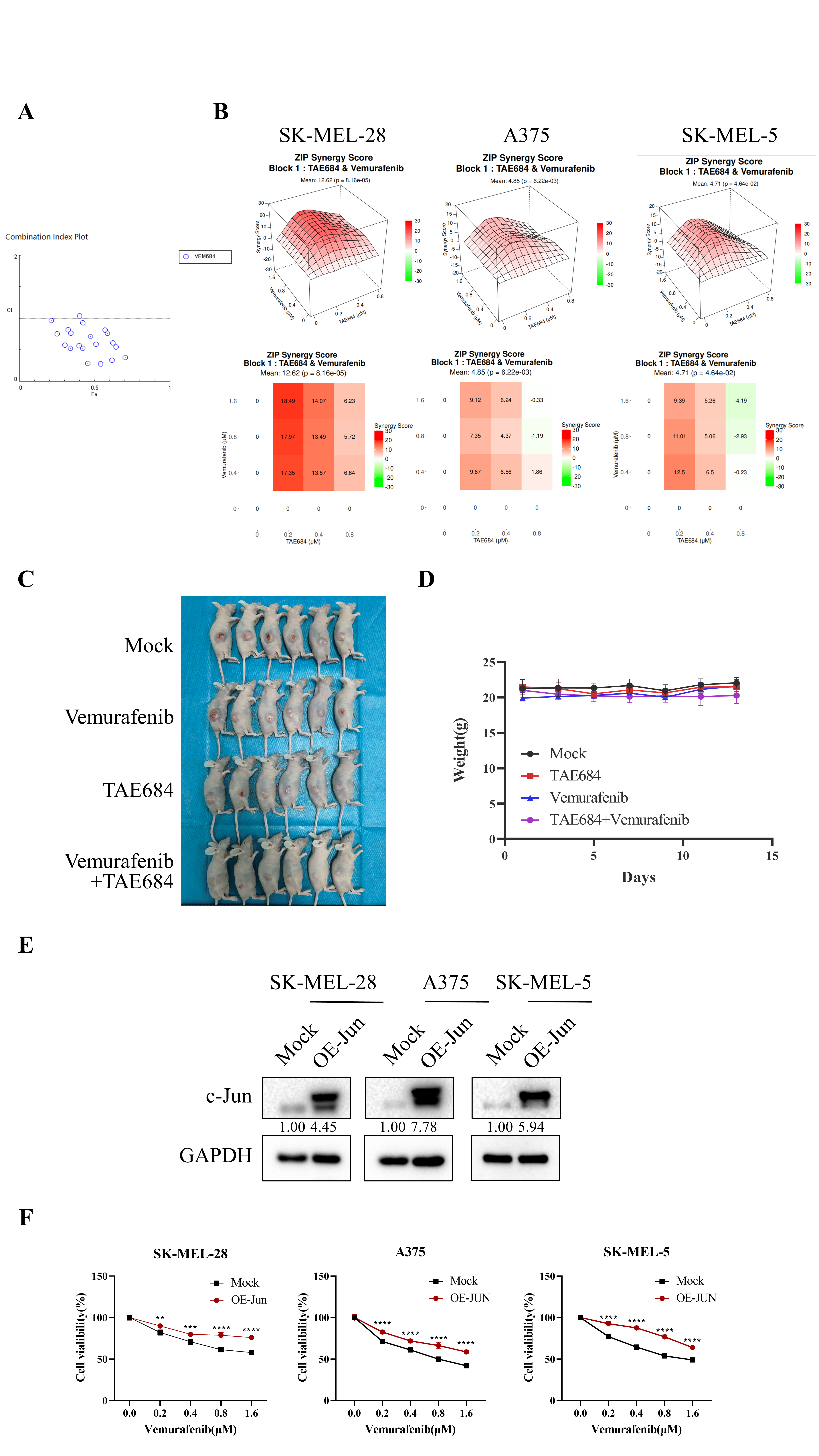

Supplement: Supplementary file 9 — Figure S9 [file 41419_2025_8090_MOESM9_ESM.tif]
