# Supplementary material for: Fyn inhibition by TAE684: A synergistic strategy to suppress melanoma and reverse vemurafenib resistance
Source: Cell Death Dis. 2025 Nov 6;16(1):796. doi: 10.1038/s41419-025-08090-1 (PMC12592403; doi:10.1038/s41419-025-08090-1)
Supplement: Supplementary file 10 — Supplementary materials [file 41419_2025_8090_MOESM10_ESM.docx]

**Supplementary materials and methods**

**Virtual screening and molecular docking**

The crystal structure of the Fyn protein (PDB ID: 2DQ7) was downloaded from the Protein Data Bank(1). The obtained protein crystal structure was processed using the Protein Preparation Wizard module of Schrödinger software, which included protein preprocessing, regeneration of the native ligand states, optimization of H-bond assignments, protein energy minimization, and removal of waters(2). The 2D SDF structure files of nine compound libraries (Selleck, catalog numbers: L1200, L1300, L1400, L1700, L1800, L3600, L3800, L5800, L7800), along with a set of in-house modified compounds, were processed using the LigPrep module of Schrödinger to generate all possible 3D chiral conformations. The Receptor Grid Generation module in Schrödinger was used to set an appropriate enclosing box that perfectly encapsulated the original ligand binding site, and the protein's active pocket was obtained based on this. The QikProp module was used to predict all ADME/T parameter scores for the ligand compounds, yielding a total of 51 types of parameters, with primary reference to the Lipinski's Rule of Five for evaluation. Each processed ligand from the nine compound libraries was docked with the active pocket of the Fyn protein using molecular docking (HTVS, SP, and XP, with docking accuracy progressively increased). A lower score indicates a lower binding free energy between the compound and protein, indicating higher binding stability. The ligand with the lowest score was selected for MM-GBSA calculation with the active site of the Fyn protein. MM-GBSA dG Bind approximates the binding free energy of the small molecule and protein, with a lower binding free energy indicating greater stability of the ligand-protein interaction.

**Reactive oxygen species (ROS) measurement**

DCFH-DA ROS probe (4091-99-0, Solarbio Life Science, China) was used for ROS measurement according to the manufacturer's protocol. In brief, 5 × 10^4^ melanoma cells were seeded in each well of 6-well plate and rested for 2 days in complete medium with certain concentrations of TAE684; Fyn knock-down cells were seeded for 12h. Then cells were digested, washed by PBS for 3 times and incubated with 5 μM DCHF-DA in serum-free DMEM for 20 minutes. Cells were then washed by PBS the fluorescent signal was detect on flow cytometer (BD Biosciences, NJ). Data were analyzed using FlowJo.

**Cell death rescue assay**

Melanoma cells were seeded into 96-well plates at a density of 2,000 cells per well. After adherence, cells were pretreated for 2 h with the following cell death inhibitors: 20 μM Z-VAD-fmk (apoptosis inhibitor), 1 μM Ferrostatin-1 (ferroptosis inhibitor), 20 μM Necrostatin-1 (necroptosis inhibitor), or 5 μM Chloroquine (autophagy inhibitor). Subsequently, cells were co-treated with 0.8 μM TAE684 and the respective inhibitors for an additional 48 h. Cell viability was then assessed using the CCK-8 assay, and absorbance was measured at 450 nm. The relative cell viability was calculated as a percentage of the untreated control group.

**Quantitative reverse transcription-PCR analysis**

The extraction of total RNA and reverse transcription for RT-PCR were performed as described previously(3). The PCR primers used in the study are listed in Supplementary Table 1. Relative mRNA expression was calculated using the 2^-△△CT^ value.

**Supplementary Table 1. PCR primers**

| Gene name | Sequence (5’ to 3’) | Direction |
| --- | --- | --- |
| c-Jun (Human) | AACAGGTGGCACAGCTTAAAC | Forward |
| c-Jun (Human) | CAACTGCTGCGTTAGCATGAG | Reverse |
| c-Fos (Human) | GGGCAAGGTGGAACAGTTAT | Forward |
| c-Fos (Human) | CGCTTGGAGTGTATCAGTCAG | Reverse |
| GAPDH (Human) | CTCTGCTCCTCCTGTTCGAC | Forward |
| GAPDH (Human) | GCCCAATACGACCAAATCC | Reverse |

**ADP-glo kinase assay**

The ADP-Glo™ Kinase Assay (V6930, Promega, WI, USA) was performed to evaluate the inhibitory effect of TAE684 on Fyn kinase activity. The kinase buffer contained 40mM Tris-HCl (pH 8.5), 0.1mg/mL BSA, 20mM MgCl₂, 20mM NaCl, 1mM TECP, and 4% DMSO. TAE684 was serially diluted in kinase buffer from 800nM using a threefold dilution series (12 concentrations in total). Each dilution (5μL) was incubated with 1μg of active Fyn kinase (ab84696, Abcam) at room temperature for 30 min. A substrate mixture containing 20 μM ATP, peptide substrates, and kinase buffer was prepared. Equal volumes of the pre-treated Fyn kinase solution and substrate mixture were combined and incubated at room temperature for 60 min. The reaction (5μL) was then transferred to an opaque white 384-well plate (#3824, Corning, NY, USA), followed by the sequential addition of 5μL ADP-Glo reagent (40 min incubation) and 10μL detection buffer (30–60 min incubation in the dark). Luminescence, correlating with kinase activity, was measured using a microplate reader. Each reaction was performed in triplicate. The IC_50_ of TAE684 against Fyn kinase was determined using nonlinear regression curve fitting in GraphPad Prism 9.0.

**Electrophoretic mobility shift assay**

A total of 3μg of nuclear protein was utilized for analysis with an AP-1 kit (AP-1 IRDye 700, 829−07925; LI-COR Biosciences, NE, USA) in accordance with the manufacturer's instructions. Immunoreactions were then detected using the Bio-Rad imaging system (Bio-Rad, USA).

**Lentiviral Packaging and Cell Infection**

Lentiviral particles were produced by co-transfecting HEK293T cells with either pLKO1-shFyn plasmids or the pLVX-c-Jun plasmid (LH836318, WZ Biosciences Inc., Shandong, China), together with the packaging plasmids psPAX2 and pMD2.G, using TurboFect transfection reagent (Thermo Scientific) (4). The pLKO1-shFyn plasmids were previously constructed and validated in our laboratory, and the corresponding shRNA sequences are listed in Supplementary Table 1(4). Six hours after transfection, the medium was replaced with fresh DMEM. The virus-containing supernatants were collected at 48 and 72 h post-transfection, pooled, and stored at -80°C.

For lentiviral infection, melanoma cells were incubated with a 1:1 mixture of fresh DMEM and lentiviral supernatant, supplemented with 10 μg/mL polybrene. After overnight incubation, the medium was replaced with fresh DMEM. Cells were then subjected to selection with 2 μg/mL puromycin until all uninfected control cells had perished. Successfully infected cells (shFyn cells or c-Jun overexpression cells) were expanded and used for subsequent experiments.

**Supplementary Table 2.** **shRNA sequences**

| **Target** | **Sequence (5'-3')** |
| --- | --- |
| shFyn-1 | GGTTACATTCCCAGCAATTATCTCGAGATAATTGCTGGGAATGTAACC |
| shFyn-2 | GGCAGAAGAGTGGTACTTTGGCTCGAGCCAAAGTACCACTCTTCTGCC |
| Mock | CCTAAGGTTAAGTCGCCCTCG |

**Dual-luciferase reporter gene assay**

The following plasmids were used in the dual-luciferase reporter assay: the pGL3 basic luciferase reporter vector (E1751; Promega, Madison, WI, USA), the pGL3-AP-1 luciferase reporter vector (constructed in our laboratory), pENTR, pENTR-Fyn (constructed in our laboratory), and the pRLTK Renilla luciferase control vector (P100001; Promega). HEK293T cells were transfected with the respective plasmids according to experimental group assignments, with pRLTK included in each group as an internal control. 24 h post-transfection, cells were treated with the indicated concentrations of TAE684 for an additional 24 h. Firefly and Renilla luciferase activities were then measured using the Dual-Luciferase Reporter Assay Kit (E1910; Promega) following the manufacturer’s instructions. Luciferase activity was quantified in four replicates per condition, and the firefly luciferase signal was normalized to Renilla luciferase activity to account for transfection efficiency.

**Figure S1 Screening of potential Fyn-targeting inhibitors based on the molecular structures of amodiaquine and Fyn. A.** Chemical structure of amodiaquine. **B.** Crystal structure of Fyn protein (PDB: 2DQ7). **C.** Based on the structural features of amodiaquine and Fyn, a series of small-molecule compounds with potential Fyn-targeting activity were screened using Schrödinger software. The figure shows the compound numbers and their corresponding chemical structures.

**Figure S2 The anti-proliferative effects of small-molecule compounds FYN001-FYN036 on melanoma cells.** SK-MEL-28 cells were seeded into 96-well plates at a density of 2,000 cells per well. After cell attachment, the cells were treated with 1 μM (**A**) or 10 μM (**B**) of the respective compounds (n = 3). Cell viability was assessed after 48 h using the CCK-8 assay, and the survival rate was calculated. Data were plotted using GraphPad Prism 9.0.

**Figure S3 TAE684 suppressed the malignant phenotype of melanoma cells. A.** CCK-8 OD values at 450nm of SK-MEL-28, A375 and SK-MEL-5 cells treated with 0 µM, 0.2 µM, 0.4 µM, 0.8 µM, 1.6 µM lorlatinib at 0 h, 24 h, 48 h, and 72 h (n=6). **B.** Colony formation assay to detect the number of SK-MEL-28, A375 and SK-MEL-5 cell clones at different concentrations (0 µM, 0.2 µM, 0.4 µM) of TAE684. **C.** Wound healing assays were performed to assess the invasion ability of SK-MEL-28, A375 and SK-MEL-5 cells treated with TAE684. The photos (at 40× magnification) were captured every 24 h and analyzed by ImageJ software. **D.** In transwell assays, SK-MEL-28, A375 and SK-MEL-5 cells treated with TAE684 for 24 h and migrated across the membrane were stained with crystal violet and imaged at 100× magnification. **E.** Immunohistochemistry staining of Ki67 (1:400) in xenografted melanoma mouse mode tissues as described in section Materials and Methods. Representative images were taken and shown. All data were presented as the mean ± SD.

**Figure S4 TAE684 induces apoptosis and cell cycle arrest in melanoma cells. A.** Apoptosis was determined by flow cytometry with Annexin V and PI double staining in SK-MEL-28, A375 and SK-MEL-5 cells (n=3). **B.** After treating with a series of concentrations of TAE684, SK-MEL-28, A375 and SK-MEL-5 cells were collected for immunoblotting. Indicator proteins of apoptosis were detected by specific antibodies and GAPDH was used as an internal control. **C.** Cell cycle distribution was detected by flow cytometry treated with TAE684 (0 μM, 0.8 μM, 1.6 μM) for 48 h in melanoma cells according to the manufacturer’s instruction. All data were presented as the mean ± SD. The significance of differences was evaluated using one-way ANOVA. *p < 0.05; *** p < 0.001; ****p < 0.0001.

**Figure S5 Effects of cell death inhibitors on the viability of melanoma cells treated with TAE684.** Melanoma cells were treated with apoptosis inhibitor Z-VAD-fmk (Z-VAD), ferroptosis inhibitor Ferrostatin-1 (Fer-1), necroptosis inhibitor Necrostatin-1 (Nec-1), or autophagy inhibitor chloroquine (CQ). For monotherapy groups, cells were treated with either 0.4 μM TAE684 or the indicated cell death inhibitors alone for 48 hours. For combination groups, cells were pretreated with the indicated inhibitors for 2 hours, followed by co-incubation with 0.4 μM TAE684 for an additional 48 hours. Cell viability was assessed using the CCK-8 assay. All data were presented as the mean ± SD. The significance of differences was evaluated using one-way ANOVA. *p < 0.05; ****p < 0.0001.

**Figure S6 Knockdown of Fyn induces G2/M cell cycle arrest in melanoma cells.** Cell cycle distribution was detected by flow cytometry in SK-MEL-28, A375 and SK-MEL-5 Fyn-knockdown cells according to the manufacture’s instruction.

**Figure S7 Targeting Fyn results in the down-regulation of c-Jun and c-Fos transcription. A-B.** The mRNA expression of c-Jun and c-Fos in SK-MEL-28, A375 and SK-MEL-5 cells was measured by RT-PCR. Total RNA was extracted from the cells after treated with indicated concentrations of TAE684 for 48 h **(A)** or knockdown of Fyn **(B).**All data were presented as the mean (n=3) ± SD. The significance of differences was evaluated using one-way ANOVA. **p < 0.01; *** p < 0.001; ****p < 0.0001.

**Figure S8 TAE684 inhibits the malignant phenotype of vemurafenib-resistant melanoma cells by mediating DNA damage and down-regulating the AP-1 signaling pathway. A.** Colony formation assay to detect the number of RA cell clones at different concentrations (0 μM, 0.2 μM, 0.4 µM) of TAE684. The clones were stained with crystal violet and counted with image J software. **B.** Wound healing assays were performed to assess the invasion ability of RA treated with different concentrations (0 μM, 0.2 μM, 0.4 µM) of TAE684. The photos were captured every 24 h and analyzed by ImageJ software. **C.** In transwell assays, RA treated with TAE684 and migrated across the membrane were stained with crystal violet and imaged at 100× magnification. **D-E.** The mRNA expression of c-Jun and c-Fos in RA cells was measured by RT-PCR. Total RNA was extracted from the cells after treated with indicated concentrations of TAE684 for 48 h **(D)** or knockdown of Fyn **(E)** (n=3)**. F.** Cell cycle distribution was detected by flow cytometry in RA Fyn-knockdown cells according to the manufacturer’s instructions. All data were presented as the mean ± SD. The significance of differences was evaluated using one-way ANOVA. *p < 0.05; **p < 0.01; ****p < 0.0001.

**Figure S9 Combination of TAE684 and vemurafenib as an effective strategy for both parental melanoma cells and vemurafenib-resistant melanoma.** **A.** The combination index values were determined by Compusyn software for all of the combined treatments. A CI < 1 indicates synergy for a compound pair. **B.** Synergy between TAE684 and vemurafenib in parental melanoma cells was analyzed using the SynergyFinder application. The upper panels visualize the average synergy score of the two drugs, while the lower panels detail the ZIP scores for different concentration combinations. **C-D.** RA cells were injected into nude mice to establish subcutaneous xenografts. Once tumors were palpable, the mice were randomized for oral gavage of 5mg/kg TAE684, 10mg/kg vemurafenib or 5mg/kg TAE684+10mg/kg vemurafenib each day. Body weights **(D)** were measured every two days. **E.** SK-MEL-28, A375 and SK-MEL-5 cells were collected for immunoblotting after infected with lentivirus (vector control) or c-Jun lentivirus. C-Jun was detected by specific antibodies and GAPDH was used as an internal control (n=6). **F.** SK-MEL-28, A375 and SK-MEL-5 cells (vector control group and c-Jun overexpression group) were seeded into 96-well plates and treated with indicated concentrations of vemurafenib for 48 h, and cell viability was tested by CCK-8 kit (n=5). All data were presented as the mean ± SD. The significance of differences was evaluated using one-way ANOVA. **p < 0.01; *** p < 0.001; ****p < 0.0001.

References:

1. Berman HM, Westbrook J, Feng Z, Gilliland G, Bhat TN, Weissig H, et al. The Protein Data Bank. Nucleic Acids Res. 2000;28(1):235-42.

2. Sastry GM, Adzhigirey M, Day T, Annabhimoju R, Sherman W. Protein and ligand preparation: parameters, protocols, and influence on virtual screening enrichments. J Comput Aided Mol Des. 2013;27(3):221-34.

3. Guo Y, Zhang X, Zeng W, Zhang J, Cai L, Wu Z, et al. TRAF6 Activates Fibroblasts to Cancer-Associated Fibroblasts through FGF19 in Tumor Microenvironment to Benefit the Malignant Phenotype of Melanoma Cells. J Invest Dermatol. 2020;140(11):2268-79.e11.

4. Zhang X, Huang Z, Guo Y, Xiao T, Tang L, Zhao S, et al. The phosphorylation of CD147 by Fyn plays a critical role for melanoma cells growth and metastasis. Oncogene. 2020;39(21):4183-97.
