# Supplementary material for: Fyn inhibition by TAE684: A synergistic strategy to suppress melanoma and reverse vemurafenib resistance
Source: Cell Death Dis. 2025 Nov 6;16(1):796. doi: 10.1038/s41419-025-08090-1 (PMC12592403; doi:10.1038/s41419-025-08090-1)
Supplement: Supplementary file 11 — Full Length Western Blot [file 41419_2025_8090_MOESM11_ESM.pdf]

Figure 1

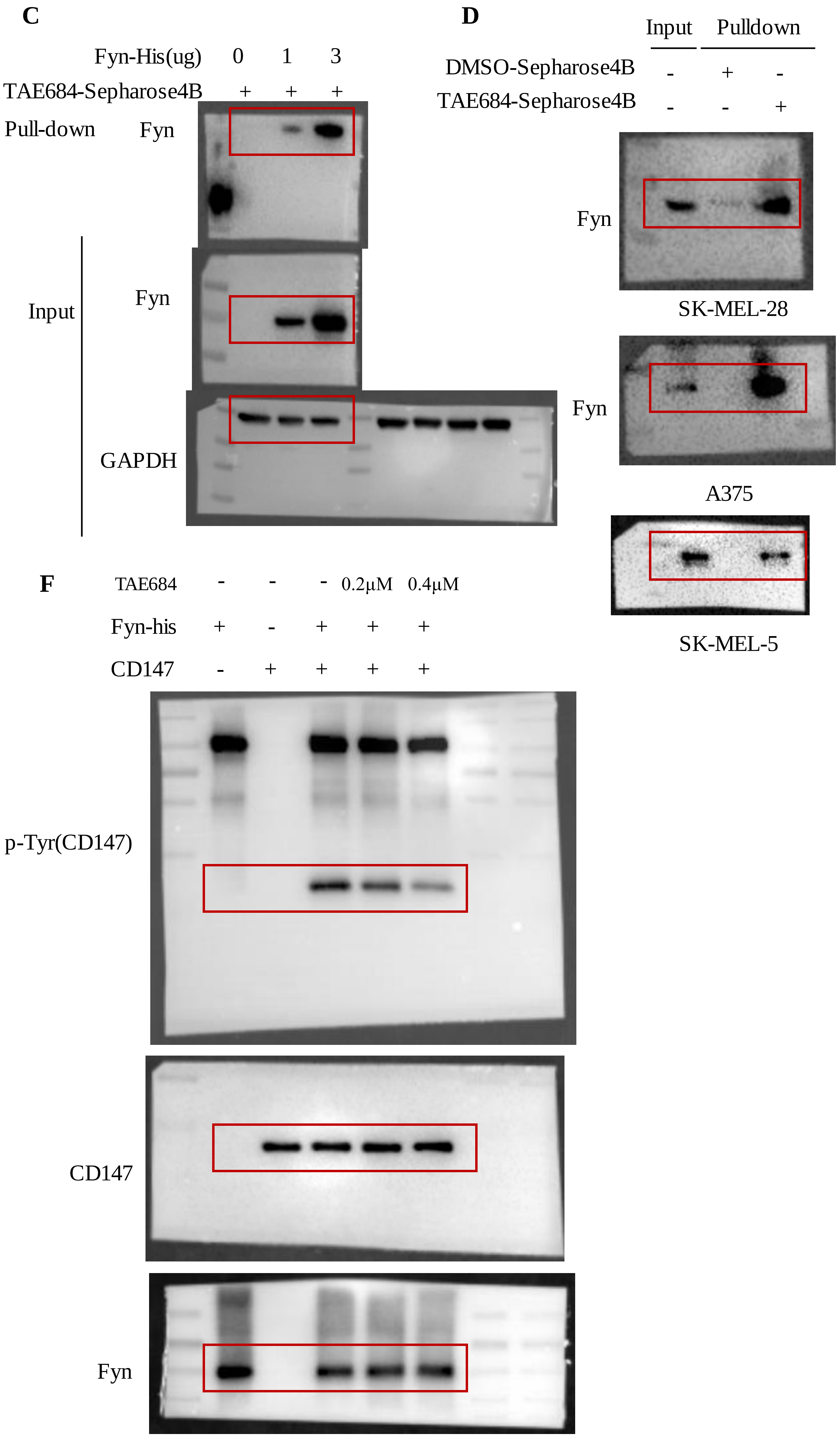

C
Fyn-His(ug) 0 1 3
TAE684-Sepharose4B + + +
Pull-down Fyn
Input Fyn
GAPDH
D
Input Pulldown
DMSO-Sepharose4B - + -
TAE684-Sepharose4B - - +
Fyn
SK-MEL-28
Fyn
A375
SK-MEL-5
F
TAE684 - - - 0.2μM 0.4μM
Fyn-his + - + + +
CD147 - + + + +
p-Tyr(CD147)
CD147
Fyn

Figure 3

B

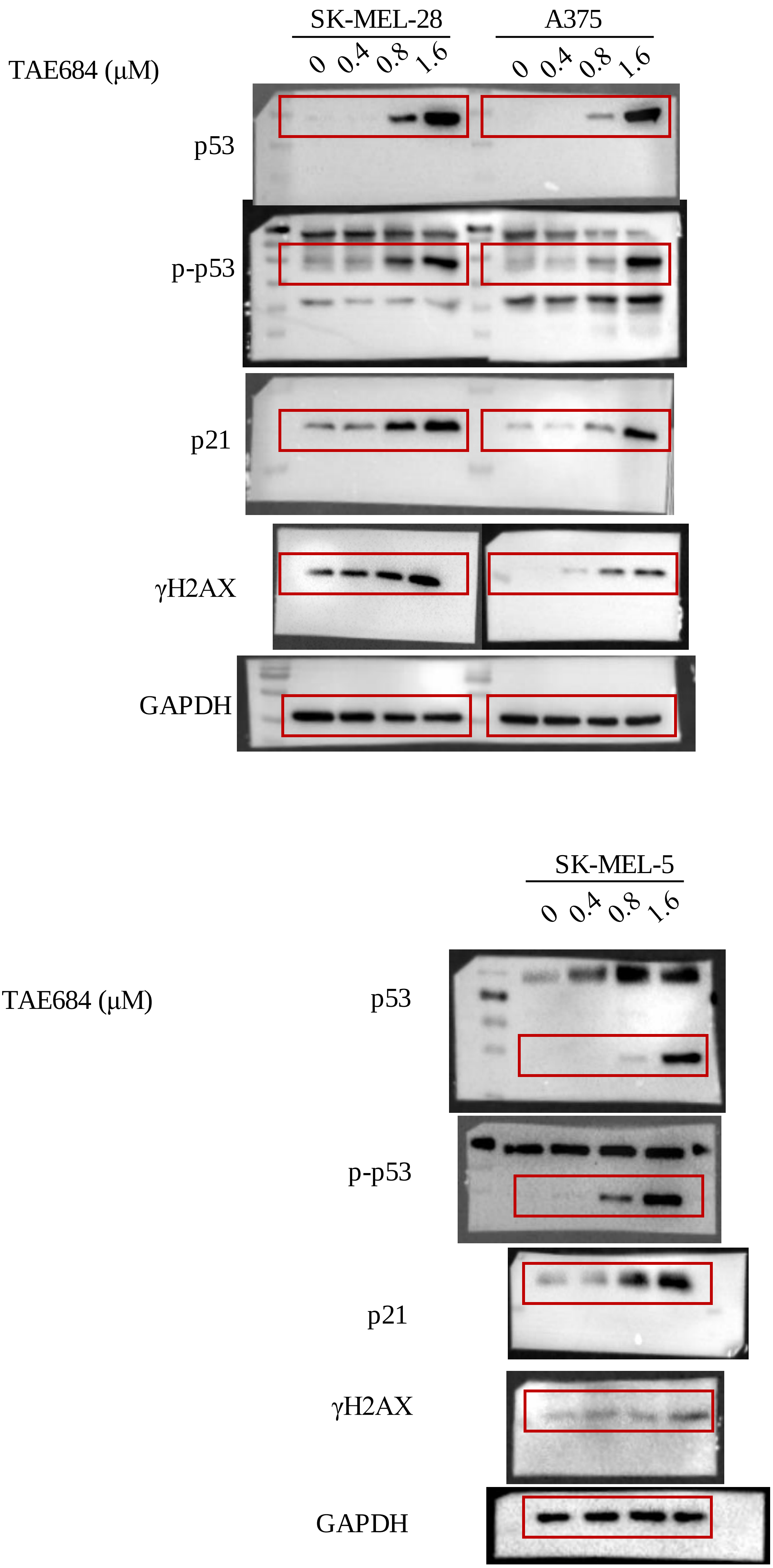
SK-MEL-28
A375
TAE684 (μM)
0 0.4 0.8 1.6
0 0.4 0.8 1.6
p53
p-p53
p21
γH2AX
GAPDH
SK-MEL-5
0 0.4 0.8 1.6
TAE684 (μM)
p53
p-p53
p21
γH2AX
GAPDH

Figure S4

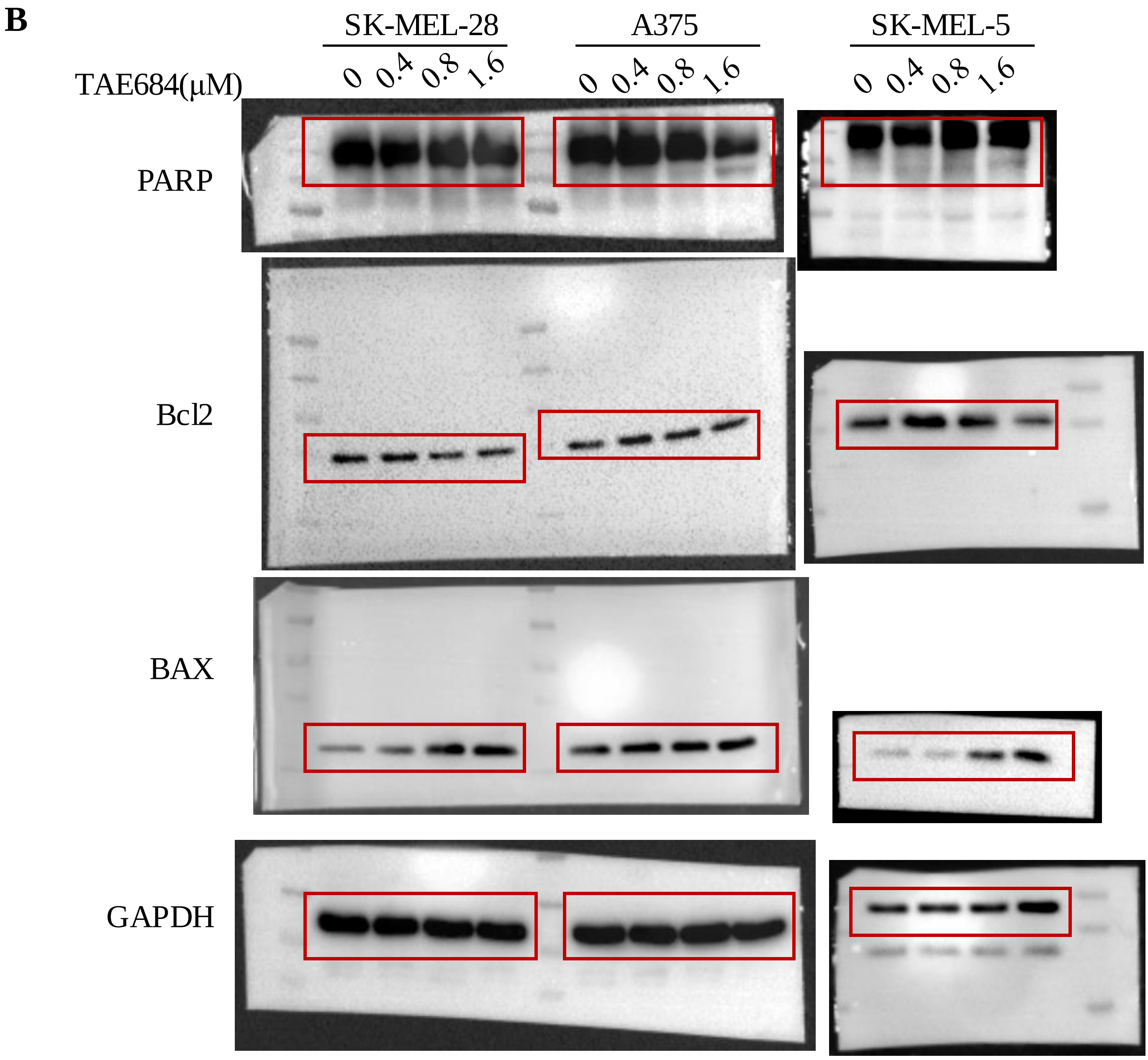
B
SK-MEL-28
A375
SK-MEL-5
TAE684(μM)
0 0.4 0.8 1.6
0 0.4 0.8 1.6
0 0.4 0.8 1.6
PARP
Bcl2
BAX
GAPDH

Figure 4

A

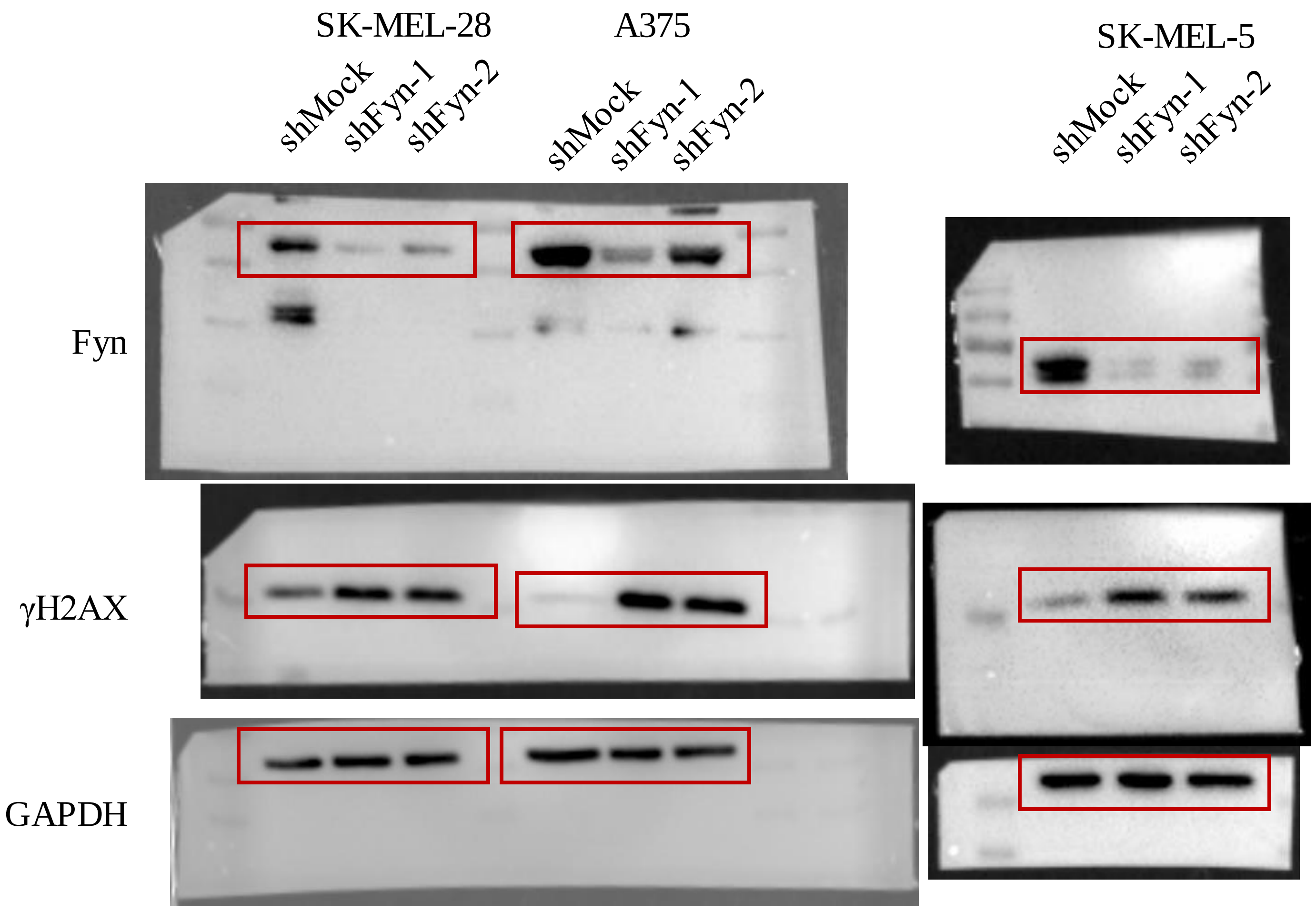
SK-MEL-28
A375
SK-MEL-5
shMock
shFyn-1
shFyn-2
shMock
shFyn-1
shFyn-2
shMock
shFyn-1
shFyn-2
Fyn
γH2AX
GAPDH

Figure 5

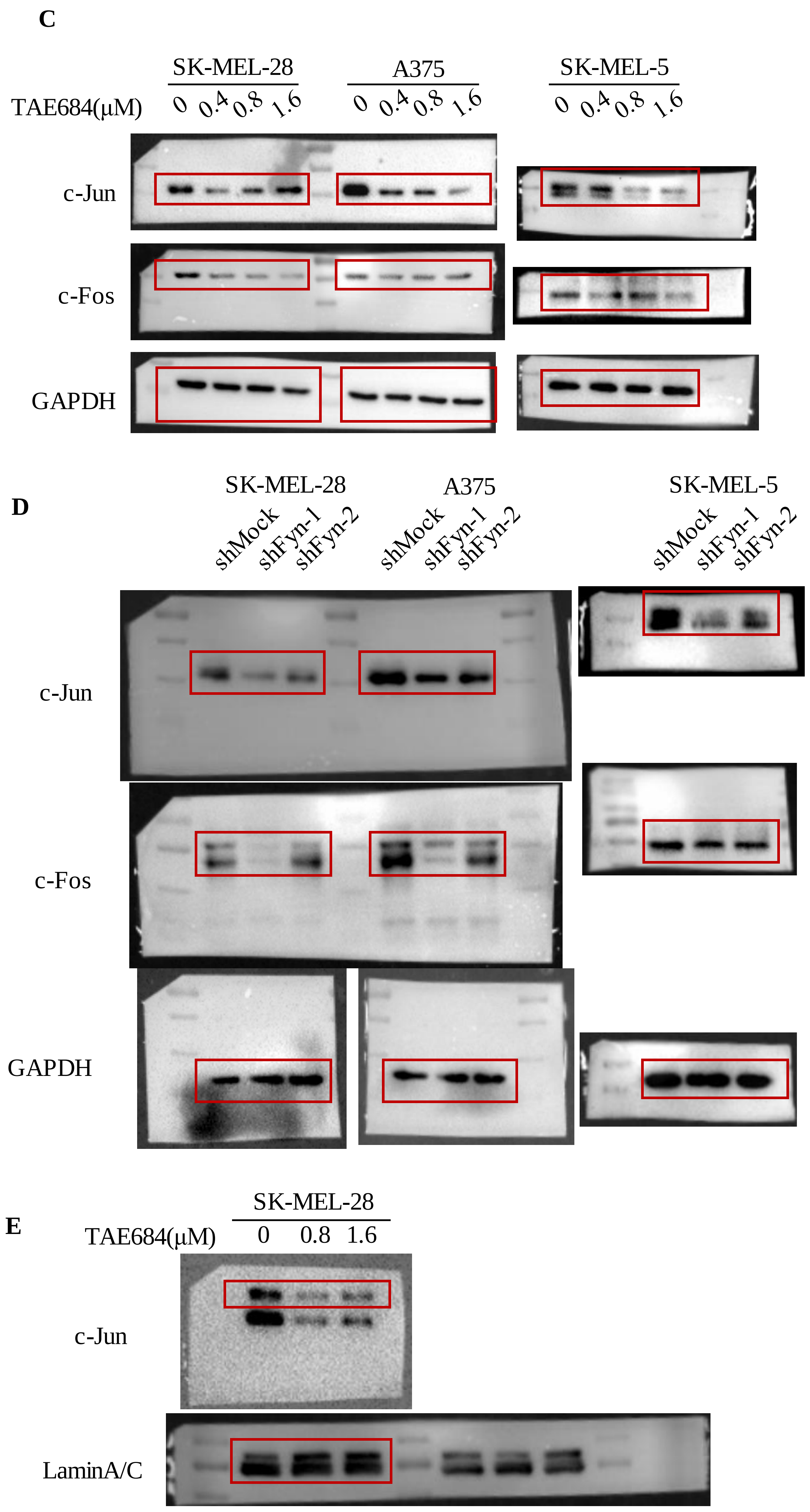
C
SK-MEL-28
A375
SK-MEL-5
TAE684(μM)
0 0.4 0.8 1.6
0 0.4 0.8 1.6
0 0.4 0.8 1.6
c-Jun
c-Fos
GAPDH
D
SK-MEL-28
A375
SK-MEL-5
shMock
shFyn-1
shFyn-2
shMock
shFyn-1
shFyn-2
shMock
shFyn-1
shFyn-2
c-Jun
c-Fos
GAPDH
E
SK-MEL-28
TAE684(μM)
0 0.8 1.6
c-Jun
LaminA/C

Figure 6

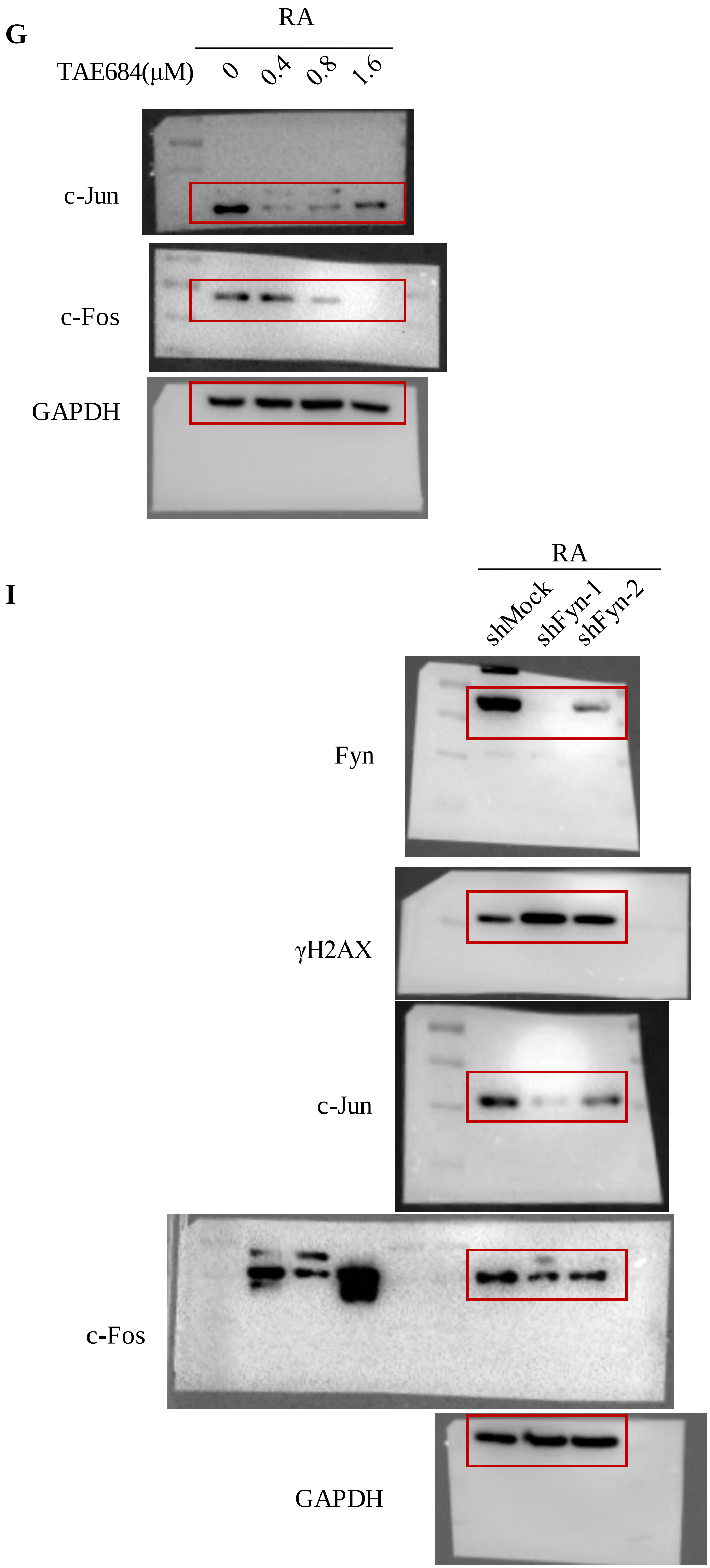
G
RA
TAE684(μM)
0
0.4
0.8
1.6
c-Jun
c-Fos
GAPDH
I
RA
shMock
shFyn-1
shFyn-2
Fyn
γH2AX
c-Jun
c-Fos
GAPDH

Figure 7

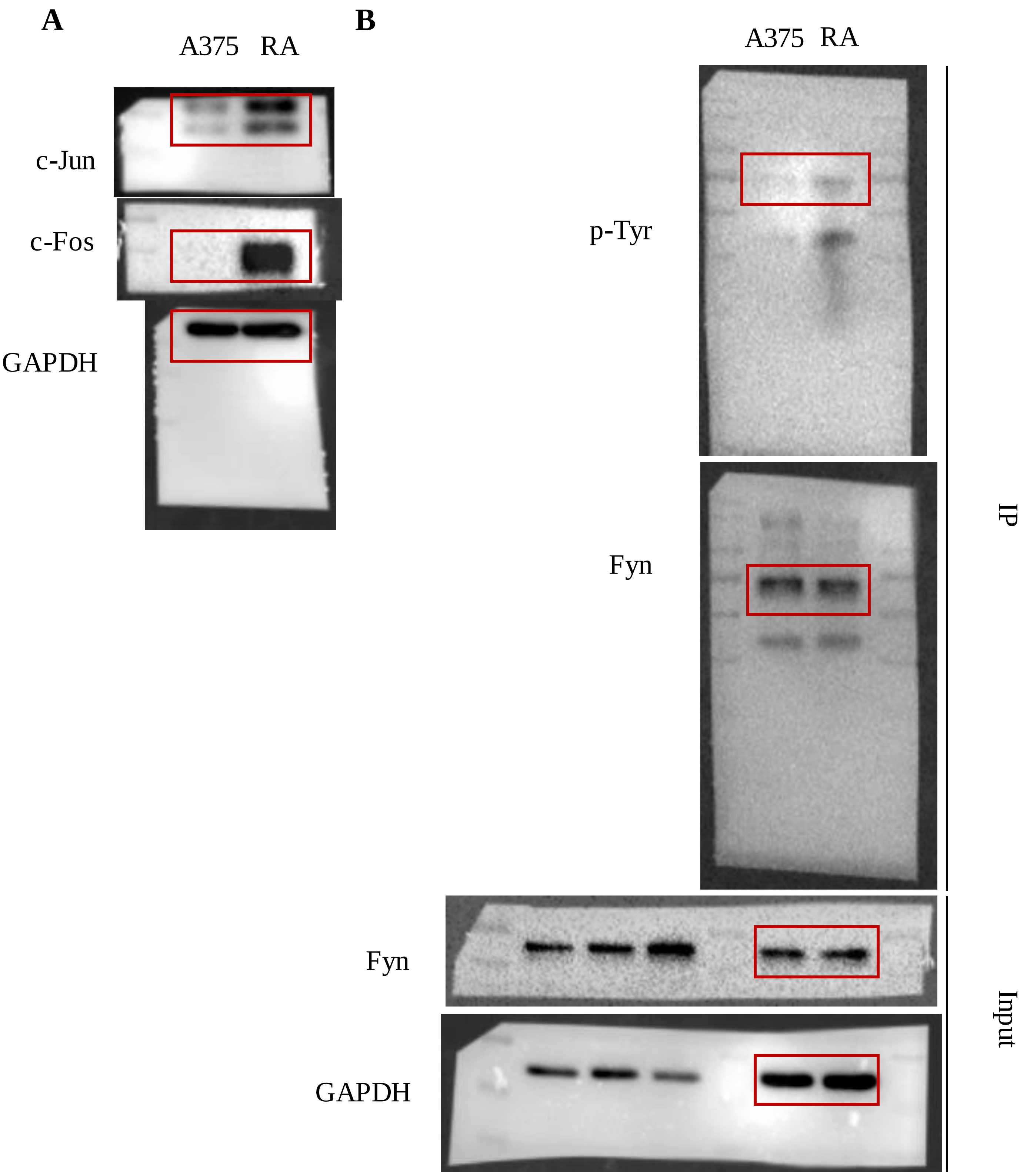

Figure S9

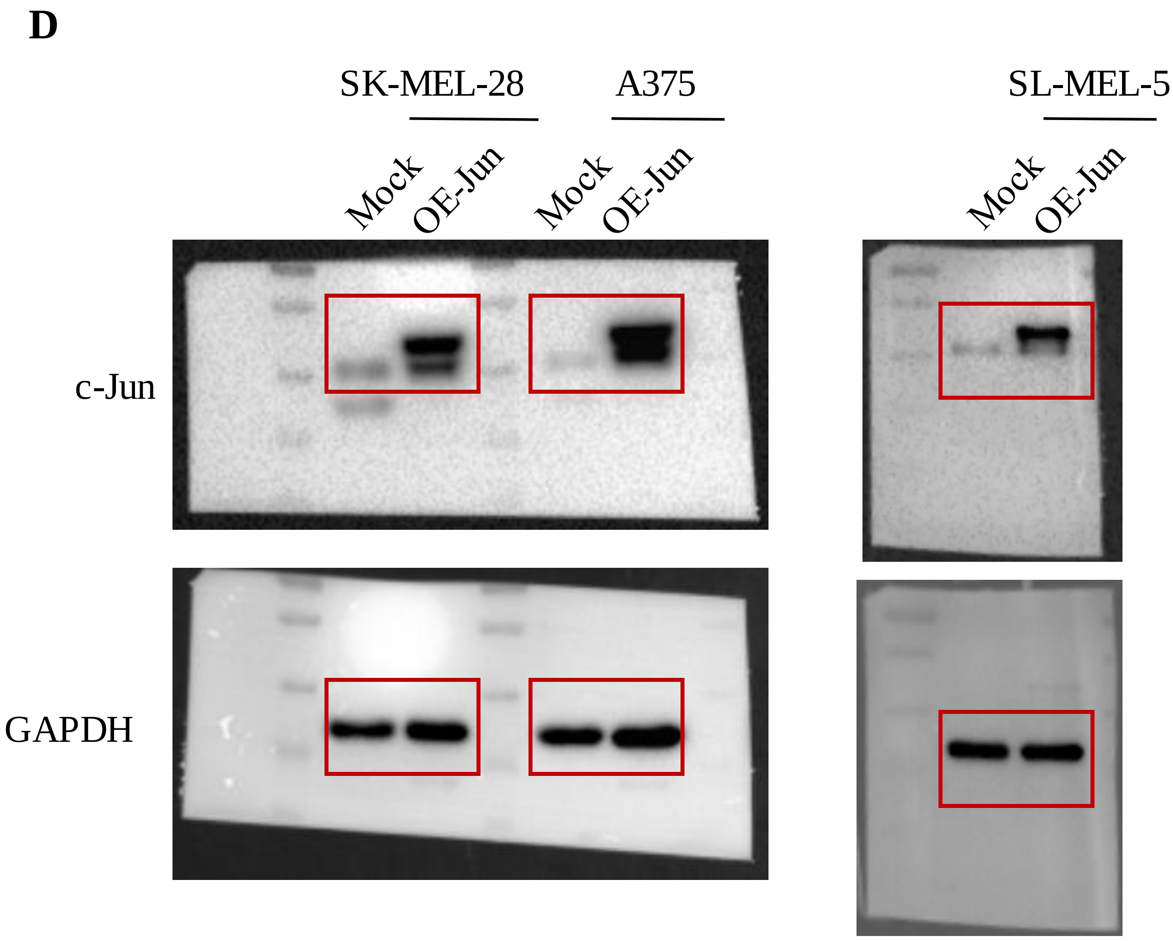
D
SK-MEL-28
A375
SL-MEL-5
Mock
OE-Jun
Mock
OE-Jun
Mock
OE-Jun
c-Jun
GAPDH
